# Photooxidation of atrazine and its influence on disinfection byproducts formation during post-chlorination: effect of solution pH and mechanism

**DOI:** 10.1038/s41598-020-77006-0

**Published:** 2020-11-23

**Authors:** Yucan Liu, Kai Zhu, Huayu Zhu, Min Zhao, Lihua Huang, Bin Dong, Qianjin Liu

**Affiliations:** 1 School of Civil Engineering, Yantai University, Yantai, 264005 China; 2 Shandong Provincial Key Laboratory of Water and Soil Conservation and Environmental Protection, College of Resources and Environment, Linyi University, Linyi, 276000 China; 3 School of Chemistry and Chemical Engineering, Linyi University, Linyi, 276000 China

**Keywords:** Photocatalysis, Environmental sciences, Pollution remediation

## Abstract

Partial photooxidation of micropollutants may lead to various degradation intermediates, obviously affecting disinfection byproducts (DBPs) formation during the post-chlorination process. The photooxidation of atrazine (ATZ) in aqueous solutions with low-pressure mercury UV lamps in UV, UV/H_2_O_2_ and UV/TiO_2_ treatment system and the formation of chlorinated disinfection byproducts (DBPs) during subsequent chlorination processes including dichloroacetic acid (DCAA), trichloroacetic acid (TCAA), 1,1,1-trichloro-2-propanone (TCP), trichloromethane (TCM) and chloropicrin (CHP) were investigated in this study. The effect of solution pH on the oxidation pathway of ATZ in three UV photooxidation treatment process and the impact of photooxidation on the DBPs formations were assessed. Based on UPLC-ESI–MS/MS analyses, identification of main oxidation intermediates was performed and the plausible degradation pathways of ATZ in photooxidation system were proposed, indicating that photooxidation of ATZ in UV/H_2_O_2_ and UV/TiO_2_ process system was significantly pH-dependent processes. Dichloroacetic acid (DCAA), trichloroacetic acid (TCAA), 1,1,1-trichloro-2-propanone (TCP), trichloromethane (TCM) and chloropicrin (CHP) were detected in photooxidized ATZ solutions. Compared to the other three DBPs, TCM and TCP were the main DBPs formed. The DBPs formations were greatly promoted in oxidized ATZ solutions. Solution pH and UV irradiation time exhibited obvious impact on the DBPs formation on the basis of DBP species. The variation tendency of DBPs observed relates to the combustion of ATZ in photooxidation system and the production oxidation intermediates.

## Introduction

Pesticides have been widely used for pests control and kill broadleaf weeds in agriculture, forestry and animal husbandry, owing to the proper biological toxicity and long half-life^1^. Due to the enormous application amounts and special molecular structures, pesticides can be transported to natural waters easily and has been detected in rivers, lakes, underground waters and sediments frequently^2–4^.

Atrazine (2-chloro-4-ethylamino-6-isopropylamino-*s*-triazine, ATZ), the most widely used pesticides for the control of weeds due to its excellent performance, is commonly detected in natural waters^5^. ATZ is also considered as an environmental hormone that induces the complete feminization, posing potential adverse effect to human health. Furthermore, trace of ATZ residue (μg/L or ng/L) in water can cause carcinogenic, teratogenetic and mutagenic effect^6^. Because of the poor efficiency of conventional water treatment processes in removing the ATZ residue, this contaminant can react with chlorine during disinfection process forming various disinfection byproducts (DBPs)^7^. Therefore, ATZ would be an important precursor of DBPs during drinking water treatment and it is meaningful to assess the potential of ATZ to produce DBPs.

Recently, chemical pre-oxidation methods including photolysis and advanced oxidation processes (AOPs) have been applied to drinking water treatment with a lot of trace organic pollutants^8^. Apart from physical methods, chemical oxidation of organics includes the cleavage chemical bonds and damage of molecular structures. Among these chemical pre-oxidation methods, photooxidation is considered as the most environmentally friendly and safe technology. The most popular photooxidation technologies are UV radiation, UV radiation with H_2_O_2_ (UV/H_2_O_2_) and UV radiation with TiO_2_ (UV/TiO_2_)^9–11^. The simplest photooxidation technology is UV radiation, the degradation of organics during sole-UV process is induced by the absorption of UV photons and direct photolysis^12^. When UV radiation is conducted along with H_2_O_2_ or TiO_2_, the processes are called UV-AOPs^13,14^. The UV-AOPs with H_2_O_2_ or TiO_2_ in solution originate the production of hydroxyl radicals (·OH), the most important radical in AOPs, in the bulk. This free radical, with standard reduction potential of 2.8 V/SHE, is the second strongest oxidizing agent after fluorine^15^. The generation of ·OH in solution promotes the oxidation rate of organic contaminants, producing large amounts of intermediates.

However, complete mineralization of organic contaminants to water and carbon dioxide generally demands relatively long reaction time and more chemicals consumption^16^. It is unprocurable to obtain complete mineralization of organics in actual water treatment process owing to the limits of reaction time and water treatment cost. Hence, oxidation products may be present in water prior to the chlorination reaction. These oxidation intermediates may be easier to react with chlorine than the precursor themselves during chlorination process^17–20^. Surprisingly, relevant papers focus on this topic are lacking, particularly regarding the role of solution pH and oxidation intermediates. Despite the level of individual organic contaminant is very low, the total contributions of contaminants to the formation of DBPs may not be ignorable.

The object of present study is to evaluate and analyze the effect of photolysis intermediates on the formation of DBPs from UV, UV/H_2_O_2_ or UV/TiO_2_ oxidized ATZ aqueous solutions following chlorination. The formation of dichloroacetic acid (DCAA), trichloroacetic acid (TCAA), 1,1,1-trichloro-2-propanone (TCP), trichloromethane (TCM) and chloropicrin (CHP) during chlorination process is analyzed by ultra-performance liquid chromatography-electrospray ionization mode-triple quadrupole mass spectrometry (UPLC-ESI–MS/MS) and a 7890A gas chromatograph fitted with a 7000A triple quadrupole mass spectrometer (GC-QqQ-MS/MS). The effects of solution pH during photooxidation processes on DBPs formation were also evaluated.

## Materials and methods

### Materials

ATZ (> 97%) was provided by TCI (Shanghai) Development Co., Ltd. (Shanghai, China) and used without further purification. Sulfuric acid (guarantee reagent), sodium hydroxide (analytical reagent), ascorbic acid (analytical reagent) and sodium hypochlorite (5% effective chlorine) were purchased from Sinopharm Chemical Reagent Co., Ltd. (Shanghai, China). The following standard solutions, HANs standard solution US EPA 511B (CHP and TCP, 2000 μg/mL of each in acetone), HAAs mixed standard solution (DCAA and TCAA, 2000 μg/mL of each in MTBE) and THMs mixed standard solution (TCM, 2000 μg/mL in acetone), were obtained from Sigma-Aldrich Corporation (Bellefonte, PA, USA). Hydrogen peroxide (30%, w/w) and TiO_2_ (P-25, mainly in anatase form) used in photooxidation process were supplied by Sinopharm Chemical Reagent Co., Ltd. (Shanghai, China) and Evonik Degussa Co. (Dusseldorf, Germany), respectively. Ultrapure water (18.2 MΩ·cm) from Elga Purelad Ultra system (Bucks, UK) was used to prepare all working solutions.

### Photooxidation experiments

An annular vessel, which had been studied in our previous research^21^, was used as photochemical reactor. The photon flux into the working solution from the low-pressure mercury UV lamp was 1.18 × 10^−7^ Einstein/s, detected with an iodode–iodate chemical actinometer. Before the oxidation reaction, 300 mL ATZ solution (5 mg/L) was transfused into the photochemical reactor, using a magnetic stirrer to maintain reaction solution homogeneity. During photooxidation process, the solution temperature was kept at 20 ± 0.5 ºC with a thermostatic water recirculation system. In order to acquire stable output, the UV lamp was ignited for 30 min before photooxidation experiments. UV/H_2_O_2_ oxidation was conducted with H_2_O_2_ added into the working solution at 5 mg/L, and UV/TiO_2_ oxidation was performed with Degussa P25 TiO_2_ powder added into the working solution at 5 mg/L. Solution pH was maintained at 4.0, 7.0 and 10.0, respectively, with 2 mM phosphate and/or borate buffers. Samples were withdrawn during photooxidation process at certain intervals to analyze the oxidation dynamics and mechanism of ATZ.

### Chlorination processes

In chlorination experiments after photooxidation, the pH value of working solutions was first adjusted to 7.0 using 1 M sulfuric acid or 1 M sodium hydroxide. After pH adjustment, a certain amount of chlorine was added into solutions to obtain free chlorine residual concentration of 1.0 ± 0.5 mg/L after 24 h chlorination. Stirred for 30 s after addition of chlorine, 50 mL ATZ photooxidation solution was injected into an amber glass flask coupled with stopper, 40 mL ATZ photooxidation solution was transferred into head-space-free amber glass bottle fitted with caps and PTFE-lined septa. These flasks and bottles were kept in dark at 25 ºC for 24 h. Ascorbic acid solution (100 g/L, 10 μL) was injected into the solution to annihilate residual chlorine to prevent further chlorination reaction. The chlorine demand was calculated from Eq. (1). All the experiments were conducted in triplicate, and average values and standard deviations were reported.

1 $$Chlorine\,demand={Cl}_{0}-{Cl}_{24}$$

where Cl_0_ and Cl_24_ were the chlorine concentrations at initial time and reaction 24 h, respectively.

### Analytic methods

The concentration of ATZ and its oxidation products were detected using UPLC-ESI–MS/MS (Waters Corporation, Milford, MA, USA) equipped with an ACQUITY UPLC BEH C_8_ column (2.1 mm × 100 mm, 1.7 μm particle). The detailed information of operation parameters is presented in Text S1 in the Supplementary materials. The extent of ATZ removal was calculated from Eq. (2).

2 $$ATZ\,removal=\frac{{ATZ}_{0}-{ATZ}_{t}}{{ATZ}_{0}}\times 100$$

where ATZ_0_ and ATZ_t_ were the detected ATZ concentrations at initial time and reaction time t, respectively.

The concentrations of DCAA and TCAA were measured with UPLC-ESI–MS/MS system based on our previous research^22^ with detailed information of operation parameters presented in Text S2 and Supplementary Table S1. The formations of TCP, TCM and CHP during chlorination process were detected by GC-QqQ-MS/MS (Agilent Technologies, Palo Alto, CA, USA) in the multiple reaction monitoring mode^23^ with specific operation parameters in Text S3 and Supplementary Table S2.

## Results and discussion

### Effects of solution pH on ATZ removal

**Figure 1:**
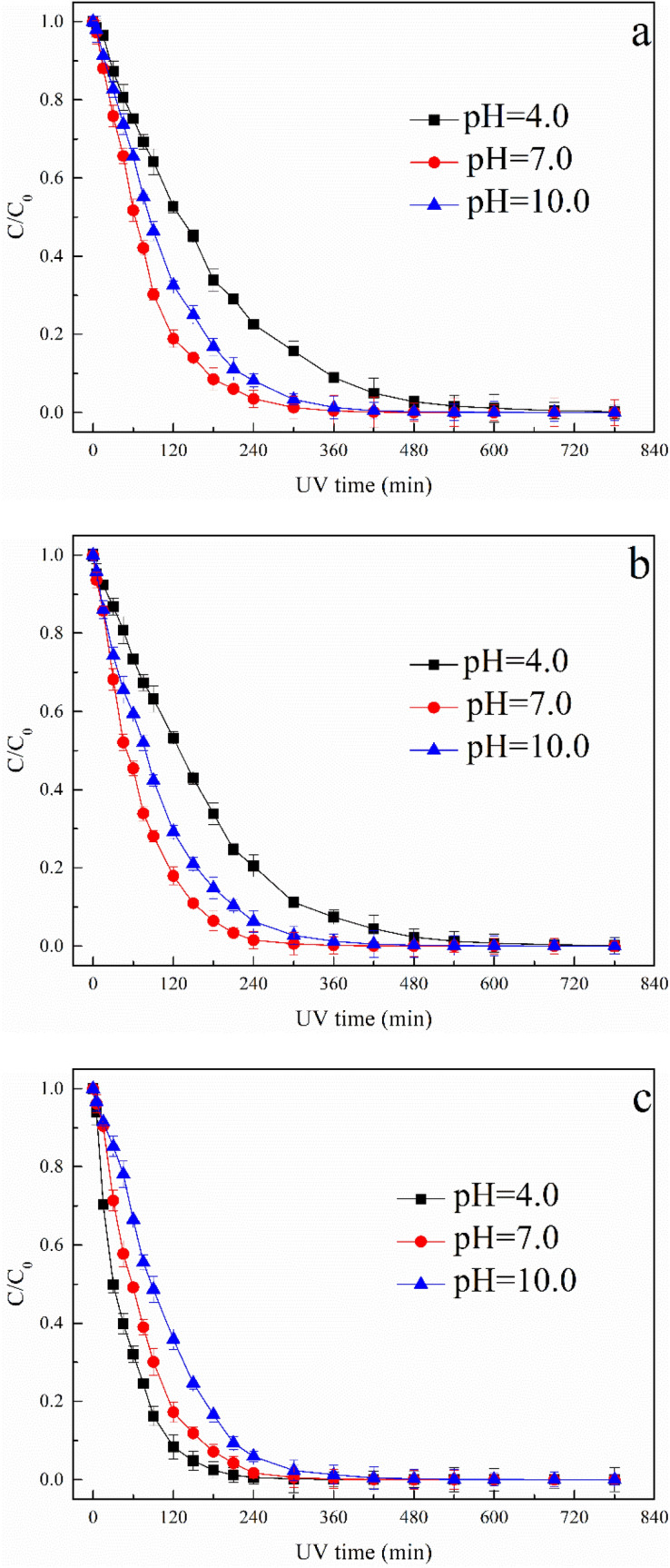
Effect of solution pH on ATZ removal: (**a**) UV process; (**b**) UV/H_2_O_2_ process; (**c**) UV/TiO_2_ process.

Different initial pH values in the range of 4–10 were chosen to evaluate the impact of solution pH on ATZ removal. The photooxidation of ATZ at different solution pH values during UV, UV/H_2_O_2_ and UV/TiO_2_ process is shown in Fig. 1.

After 240 min irradiation, the removal efficiency of ATZ during UV process reached 77.5%, 96.5% and 91.8% with solution pH values as 4.0, 7.0 and 10.0, respectively. Obviously, the removal efficiency of ATZ increased sharply with the increase of solution pH value from 4.0 to 7.0. Nevertheless, when the solution pH value further increased from 7.0 to 10.0, the removal efficiency of ATZ exhibited slight drop. The effect of solution pH on the photooxidation of ATZ is mainly due to the distribution of pH-dependent species, protonated and deprotonated ATZ species^21,24^. As solution pH increased from 4.0 to 7.0, ATZ molecules mainly exist in negative forms, which increase the electron density of *s*-triazine ring and thus promoted the indirect photodegradation of ATZ^25^. Further increased the solution pH to 10.0, the UV–Vis absorbance spectra of ATZ showed significant decreasing trend, blocking the absorption of photons^21^. During UV/H_2_O_2_ process, the removal efficiency of ATZ exhibited analogous tendency, attaining 79.5%, 98.5% and 93.7% after 240 min treatment under solution pH values of 4.0, 7.0 and 10.0, respectively. The oxidation of ATZ in UV/H_2_O_2_ system was due to the synthetic action of ·OH generated by photolysis of H_2_O_2_ and direct photolysis. The oxidation power of ·OH decreased as the increase of solution pH^26^. However, in neutral condition, the photolysis rate of ATZ is the highest. The results also indicated that addition of H_2_O_2_ during UV irradiation promoted the degradation of ATZ due to the formation of ·OH in the bulk.

During the initial stage of UV/TiO_2_ process, the removal efficiency of ATZ decreased significantly as solution pH increased from 4.0 to 10.0. The pH_pzc_ of TiO_2_ is reported in the range of 6.3–6.9 by several papers^27,28^. The surface of TiO_2_ particle would be negatively charged when pH > pH_pzc_, positively charged when pH < pH_pzc_. Meanwhile, the distribution of protonated and deprotonated ATZ species varied with solution pH, and ATZ molecules had a negative charge in acidic condition. The ATZ molecules were easily adsorbed to the surface of TiO_2_ particles in acidic condition in UV/TiO_2_ process. In order to confirm that the decay of ATZ in UV/TiO_2_ process was caused by the photooxidation only, adsorption experiment was carried out in dark using 5 mg/L TiO_2_ as adsorbent in 300 mL solution with ATZ concentration at 5 mg/L. No significant signs of adsorption were observed, indicating that TiO_2_ adsorption play no distinct role during photooxidation of ATZ in UV/TiO_2_ process (see Supplementary Fig. S1).

Supplementary Figure S2 shows the semi-log graph of the ATZ versus UV photooxidation time with different solution pH. Supplementary Tables S3–S5 exhibits the relevant apparent rate constant *k* and correlation coefficient *R*^2^. These figures and tables confirmed that the photooxidation of ATZ at different pH values during UV, UV/H_2_O_2_ and UV/TiO_2_ process followed the pseudo-first-order kinetics. Solution pH had obvious significance on the *k* value, the *k* values were 0.00779, 0.01545 and 0.01308 min^−1^ in sole-UV system with initial solution pH at 4, 7 and 10, respectively. The *k* values were 0.00842, 0.01703 and 0.01253 min^−1^ in UV/H_2_O_2_ system with initial solution pH at 4, 7 and 10, respectively. The *k* values were 0.02042, 0.01765 and 0.01289 min^−1^ in UV/TiO_2_ system with initial solution pH at 4, 7 and 10, respectively. The abovementioned tendency can be attributed to the oxidation effect of ·OH generated in UV/H_2_O_2_ and UV/TiO_2_ process. With the increase of solution pH, ATZ molecules mainly exist in negative forms, which increase the electron density of *s*-triazine ring and thus promoted the indirect photodegradation of ATZ in solution^25^. On the other hand, the generation of ·OH generated in UV/H_2_O_2_ and UV/TiO_2_ process was strikingly affected by the solution pH.

### Photooxidation intermediates and plausible mechanism

The primary intermediates of ATZ during photooxidation process were detected and identified by comparing the total ion chromatograms (TICs) of ATZ solutions before and after UV, UV/H_2_O_2_ and UV/TiO_2_ process treatment.

#### Photooxidation intermediates and degradation pathway in UV process

**Figure 2:**
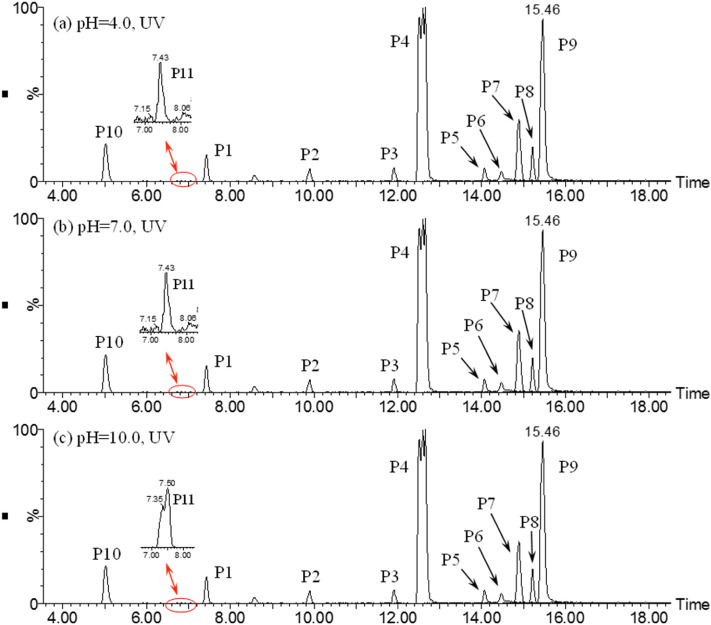
The extracted ion chromatograms (EICs) of ATZ solution after 90 min irradiation in UV process: (**a**) pH = 4.0; (**b**) pH = 7.0; **(c)** pH = 10.0.

The extracted ion chromatograms (EICs) of ATZ solutions after UV treatment are exhibited in Fig. 2. As can be seen, the influence of solution pH on ATZ degradation mechanism is negligible during UV treatment, since the EICs of ATZ solutions at pH of 4.0, 7.0 and 10.0 are almost the same. ATZ and its ten photolysis intermediates were detected, and for clear description, these peaks were labeled as P1, P2, P3, P4, P4, P5, P6, P7, P8, P9 (ATZ), P10 and P11.

MS and MS/MS spectra analyses under ESI^+^ and ESI^−^ mode were performed for identifying the molecular structures of these intermediates. The retention times and MS spectral information of the mentioned 11 intermediates are listed in Supplementary Table S6. Molecular weight (MW) of the 11 intermediates, deduced from the *m*/*z*, were used to analyze the molecular structures. Furthermore, in order to acquire further information of these intermediates, collision induced dissociation (CID) experiments were performed. Argon was used as collision gas and the collision energy of each intermediate was optimized. The precursor ions of each intermediate under daughter scan mode are shown in Supplementary Table S7, and the particular information of these fragments during MS/MS analysis are shown in Supplementary Figs. S3–S12. Molecular structure of these intermediates were deduced on the basis of MS spectra and MS/MS spectra.

In the case of P1, only detected under ESI^+^ mode, the *m*/*z* of main fragment in MS spectra was 154. Under ESI^+^ mode, H^+^ added to P1 produced the positive ion, indicating the MW of P1 was 153 Da. In addition, four fragment ions (*m*/*z* 112, 85, 70 and 68) were detected in the MS/MS spectra as shown in Supplementary Fig. S3. P1 (*m*/*z* 154) lost –CH(CH_3_)_2_ (*m*/*z* 42) produced the fragment ion of *m*/*z* 112, and fragment ion of *m*/*z* 85 lost –NH_2_ generated fragment ion of *m*/*z* 70. MS/MS spectra indicated the *s*-triazine ring of P1 attached –NHCH(CH_3_)_2_ and –NH_2_.

The MW of P2 (*m*/*z* 184), detected under ESI^+^ mode, was 183 Da. P2 lost –(CH_3_)_2_ generated fragment ion of *m*/*z* 156, lost –CH(CH_3_)_2_ generated fragment ion of *m*/*z* 142, and lost –CH(CH_3_)_2_, –NHCH_3_ and –OH generated fragment ion of *m*/*z* 97, indicated the molecular structure of P2 included –NHCH(CH_3_)_2_ and –NHCH_3_.

The precursor ion of P3 (*m*/*z* 196) produced five fragment ions (*m*/*z* 145, 97, 89, 71 and 65). Based on MS, MS/MS spectra and previous researches^29,30^, the possible molecular structure of P3 was concluded.

P4 was detected under both ESI^+^ and ESI^−^ modes. P4 lost –CH(CH_3_)_2_ generated fragment ion of *m*/*z* 156, lost –CH(CH_3_)_2_ and –CH_2_CH_3_ generated fragment ion of *m*/*z* 128, and lost –NHCH(CH_3_)_2_, –CH_2_CH_3_ and –OH generated fragment ion of *m*/*z* 97 under ESI^+^ mode. Under ESI^−^ mode, P4 lost –(CH_3_)_2_ generated fragment ion of *m*/*z* 168, lost –CH(CH_3_)_2_ generated fragment ion of *m*/*z* 154, lost –NHCH(CH_3_)_2_ and –CH_3_ generated fragment ion of *m*/*z* 125, and lost –NHCH(CH_3_)_2_ and –CH_2_CH_3_ generated fragment ion of *m*/*z* 111. Based on above analysis, the molecular of P4 is shown in Supplementary Table S8.

The precursor ions of P5 and P6 were detected under both ESI^+^ and ESI^−^ modes with the same *m*/*z*. For P5, the fragment ion of *m*/*z* 156 was produced by the loss of –COCH_3_, the fragment ion of *m*/*z* 139 was produced by the loss of –NHCOCH_3_ and the fragment ion of *m*/*z* 113 was produced by the loss of –NHCOCH_3_ and –CH_2_CH_3_. For P6, the fragment ion of *m*/*z* 156 was produced by the loss of –CHCH_2_ and –OH, the fragment ion of *m*/*z* 153 was produced by –CH_3_ instead of –NHCHOCH_3_ in this position, the fragment ion of *m*/*z* 127 was produced by the loss of –NHCHCH_2_, –CH_3_ and –OH, and the fragment ion of *m*/*z* 113 was produced by the loss of –NHCHCH_2_ and –CHOHCH_3_.

P7 and P8 had same molecular ions *m*/*z* 212 under ESI^+^ mode, indicating the MWs of P7 and P8 were 211. For P7, the fragment ion of *m*/*z* 170 was produced by the loss of –CH(CH_3_)_2_, the fragment ion of *m*/*z* 128 was produced by the loss of –CH(CH_3_)_2_ and –COCH_3_. The MS/MS spectra of P8 suggested the fragment ion of *m*/*z* 182 was produced by the loss of –OCH_3_, the fragment ion of *m*/*z* 170 was produced by the loss of –CH(CH_3_)_2_, the fragment ion of *m*/*z* 142 was produced by the loss of –CH(CH_3_)_2_ and –CH_2_CH_3_, the fragment ion of *m*/*z* 128 was produced by the loss of –CH(CH_3_)_2_, –CH_2_CH_3_ and –OH.

P9 was identified as ATZ by the TIC of ATZ solution before UV oxidation. P10 was only detected under ESI^+^ mode and P11 was detected under both ESI^+^ and ESI^−^ modes. For P10, two fragment ions (*m*/*z* 81 and 72) were detected in the MS/MS spectra as shown in Supplementary Fig. S12. For P11, under ESI^+^ mode, four fragment ions (198(M + H), 220(M + Na), 395(2 M + H) and 417(2 M + Na)) were detected in the MS/MS spectra, 196(M-H) was detected under ESI^−^ mode. On the basis of the MS/MS spectra and previous study^21^, the molecular structures of P10 and P11 were deduced and presented in Supplementary Table S8.

**Figure 3:**
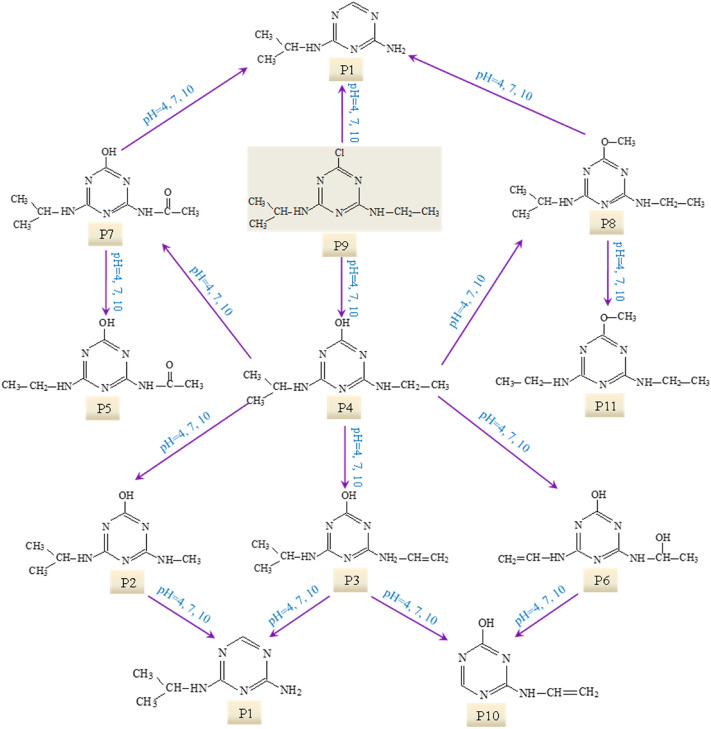
The proposed degradation pathway of ATZ in UV process.

Based on these identified photooxidation intermediates, the main reactions of ATZ in UV irradiation treatment were proposed. The dominating reaction is dechlorination–hydroxylation, replacing the Cl atom with –OH. The product of dechlorination–hydroxylation reaction included P2, P3, P4, P5, P6 and P7. Dechlorination–dealkylation reaction, including the cleavage of C–Cl, C–C and C–N bonds, produced P2. Dealkylation reaction, depriving alkyl groups from ATZ generated P1 and P10. Deamination reaction, including the removal of lateral chains connected to the –NH_2_, produced P10. Alkylic-oxidation, resulting in the formation of carbon radical compound, produced P5 and P7. Dehydrogenation–olefination, abstracted H from lateral chains of ATZ by reactive radicals, produced P3 and P6. Dechlorination–hydrogenation, resemblance to dechlorination–hydroxylation, generated P1 and P10. Dechlorination–methoxylation, initiated by small number of methanol from reserving solution of ATZ, generated P8 and P11. Dehydroxylation, containing the removal of hydroxyl from *s*-triazine and photooxidation products, formed P1. The plausible degradation pathway of ATZ in aqueous solution during direct UV irradiation process was proposed based on above analysis as shown in Fig. 3.

#### Photooxidation intermediates and degradation pathway in UV/H_2_O_2_ process

**Figure 4:**
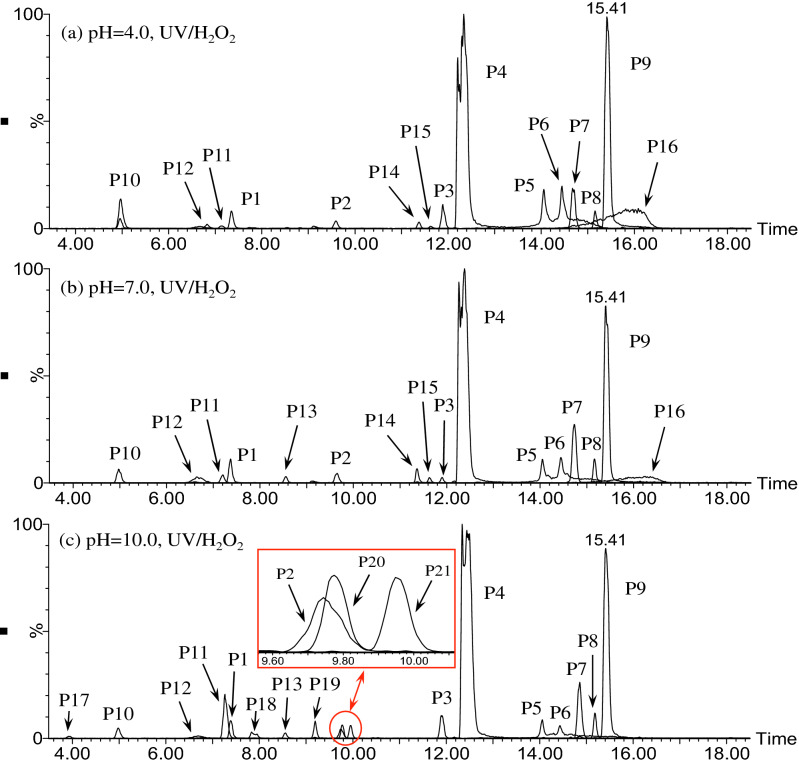
The extracted ion chromatograms (EICs) of ATZ solution after 90 min irradiation in UV/H_2_O_2_ process: (**a**) pH = 4.0; (**b**) pH = 7.0; **(c)** pH = 10.0.

The EICs of ATZ solutions after UV/H_2_O_2_ oxidation are exhibited in Fig. 4. The main photooxidation intermediates of ATZ were found to vary with solution pH. In the case of pH 4.0, ATZ and its fourteen oxidation intermediates were identified, including P1–P10, P11, P12, P14, P15 and P16. At pH 7.0, ATZ and fifteen oxidation intermediates were detected (P1–P16), including a new appeared peak (P13). As for pH 10.0, eighteen peaks were extracted, peaks of P14, P15 and P16 disappeared and five new peaks appeared (P17, P18, P19, P20 and P21). The precursor ions of P12–P21 under daughter scan mode are shown in Supplementary Table S9, and the particular information of these fragments during MS/MS analysis are shown in Supplementary Table S10 and Supplementary Figs. S13–S22. The proposed structure and chemical name of P12–P21 are shown in Supplementary Table S11.

For P12, detected under ESI^+^ mode, the MS spectra indicated its MW was 209 Da. Four fragment ions (*m*/*z* 168, 152, 123 and 115) were detected in the MS/MS spectra as shown in Supplementary Fig. S13. P12 (*m*/*z* 220) lost –COCH_3_ produced the fragment ion of *m*/*z* 168, and fragment ion of *m*/*z* 168 lost –OH generated fragment ion of *m*/*z* 152. MS/MS spectra indicated the *s*-triazine ring of P12 attached –OH, –NH(C=CH_2_)CH_3_ and –NH_2_COCH_3_. P13 only detected under ESI^+^ mode, with MW of 138 Da. P13 lost –COCH_3_ generated fragment ion of *m*/*z* 97, indicated the molecular structure of P13 included –NH_2_COCH_3_.

P14 and P15 had same molecular ions *m*/*z* 212 under ESI^+^ mode, indicating the MWs of P14 and P15 were 211. For P14, the fragment ion of *m*/*z* 184 was produced by the loss of –(CH_3_)_2_, the fragment ion of *m*/*z* 170 was produced by the loss of –CH=CH_2_ and the fragment ion of *m*/*z* 142 was produced by the loss of –(CH_3_)_2_ and –CH=CH_2_. The MS/MS spectra of P15 suggested the fragment ion of *m*/*z* 170 was produced by the loss of –CH_3_, –CH_3_ and –CH_3_, the fragment ion of *m*/*z* 103 and 86 were produced by the cleavage of *s*-triazine ring.

P16, P17 and P18 were detected under ESI^+^ mode with the same *m*/*z* 214, indicating the MWs of P16, P17 and P18 were 213. For P16, the fragment ion of *m*/*z* 173 was produced by the loss of –NHCH=CH_2_. The MS/MS spectra of P17 suggested the fragment ion of *m*/*z* 196 was produced by the loss of –OH, the fragment ion of *m*/*z* 170 was produced by the loss of –CH_3_, –CH_3_ and –OH, the fragment ion of *m*/*z* 143 was produced by the loss of –CH_3_ and –NHCH(CH_3_)_2_ and the fragment ion of *m*/*z* 129 was produced by the cleavage of *s*-triazine ring.

P19 was detected under ESI^+^ modes with MW of 195 Da. P19 generated fragment ion of *m*/*z* 154 by loss of –CH_3_ and –CH_2_CH_3_, and lost –NHC_3_H_5_ and –CH_3_ generated fragment ion of *m*/*z* 127. For P18, P20 and P21, no fragment ions were detected in MS/MS spectra. The molecular structure of P18, P20 and P21 were deduced by MWs and previous researches^21,31^.

**Figure 5:**
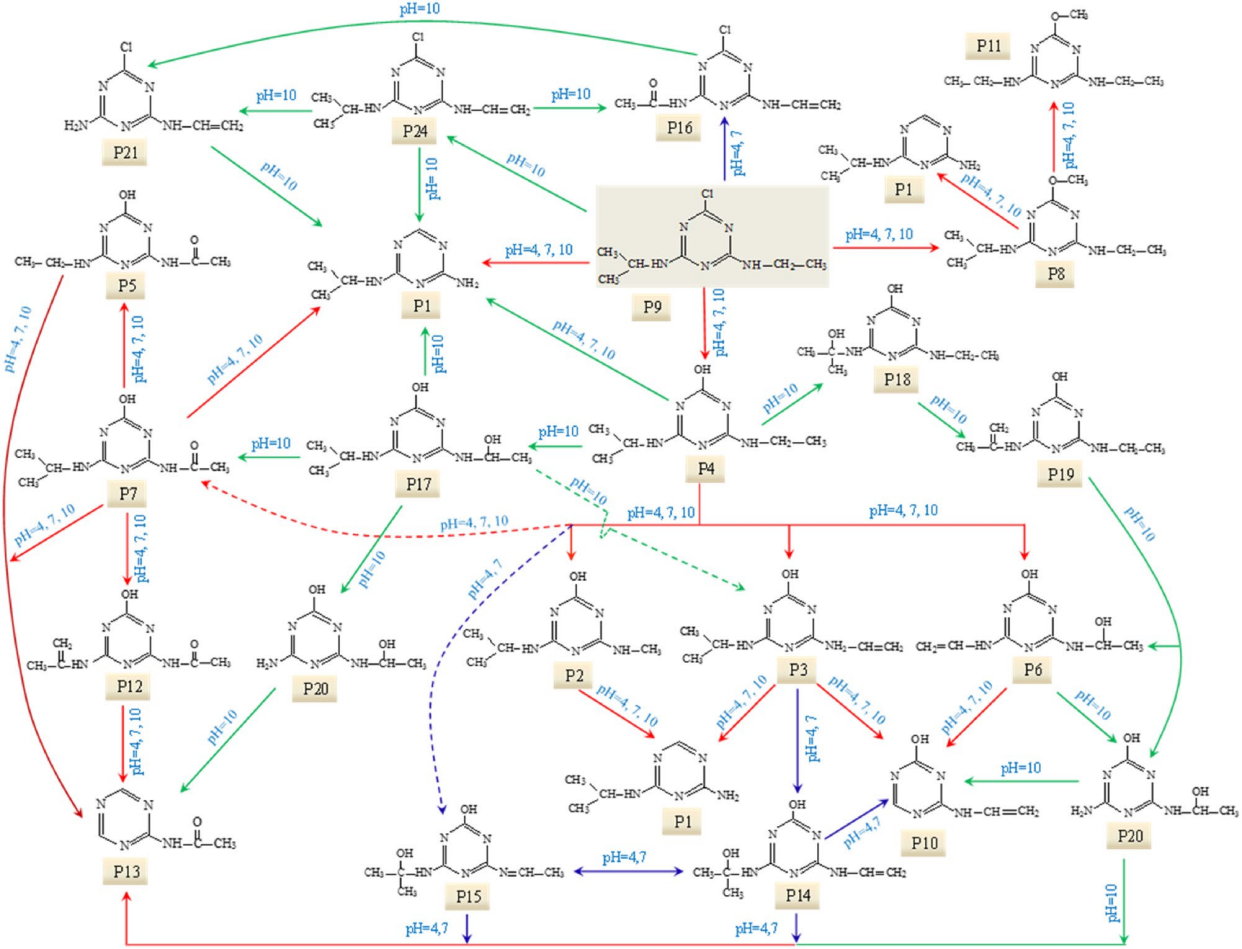
The proposed degradation pathway of ATZ in UV/H_2_O_2_ process.

Based on these identified photooxidation intermediates, the oxidation pathway of ATZ in UV/H_2_O_2_ process was proposed, as shown in Fig. 5. Apart from these intermediates detected in direct UV irradiation treatment, several new oxidation products (P12–P21) were identified in UV/H_2_O_2_ oxidation system. These intermediates were probably generated by the attack of ·OH^32–34^. Two feasible oxidation modes of ·OH onto ATZ molecular existed in UV/H_2_O_2_ system, (1) abstraction of hydrogen atom (P12, P14, P15, P16 and P21), (2) the hydroxylation attack (P14, P15, P17, P18, P19 and P20)^35,36^.

#### Photooxidation intermediates and degradation pathway in UV/TiO_2_ process

**Figure 6:**
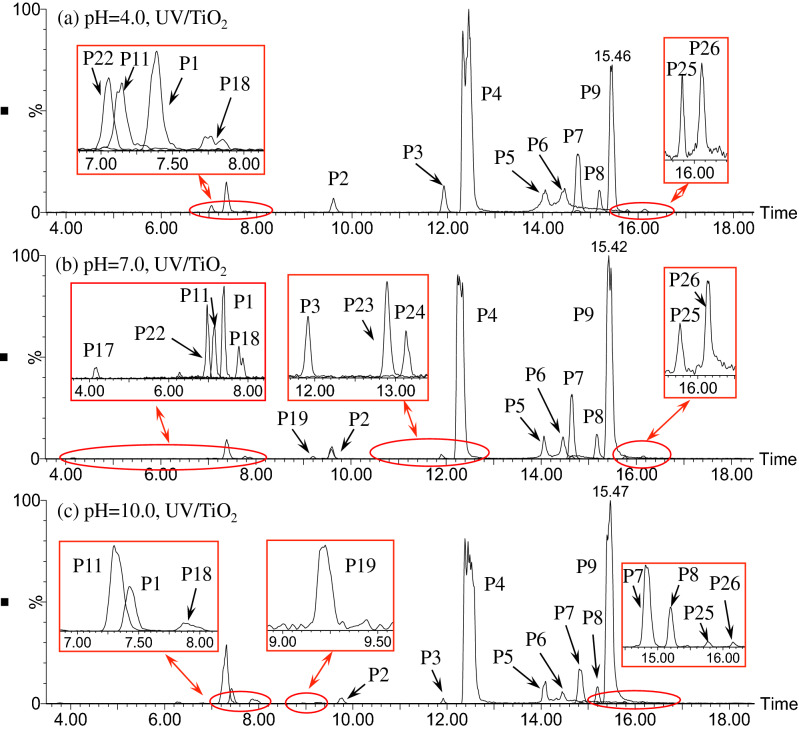
The extracted ion chromatograms (EICs) of ATZ solution after 90 min irradiation in UV/TiO_2_ process: (**a**) pH = 4.0; (**b**) pH = 7.0; **(c)** pH = 10.0.

The EICs of ATZ solutions after UV/TiO_2_ oxidation are exhibited in Fig. 6. The effect of solution pH on the oxidation pathway of ATZ in UV/TiO_2_ process was remarkable. At pH 4.0, thirteen oxidation intermediates were identified, including P1–P8, P11, P18, P22, P25 and P26. However, in case of pH 7.0, ATZ and seventeen oxidation intermediates were detected, including P1–P8, P11, P18, P22, P25, P26 and 4 new appeared peaks (P17, P19, P23 and P24). As for pH 10.0, the number of detected intermediates was changed again, thirteen peaks were extracted. Compared to pH 4.0, peak P19 replaced P22 at pH 10.0. The MS fragment information for the new intermediates of ATZ in UV/TiO_2_ process are shown in Supplementary Table S12.

**Figure 7:**
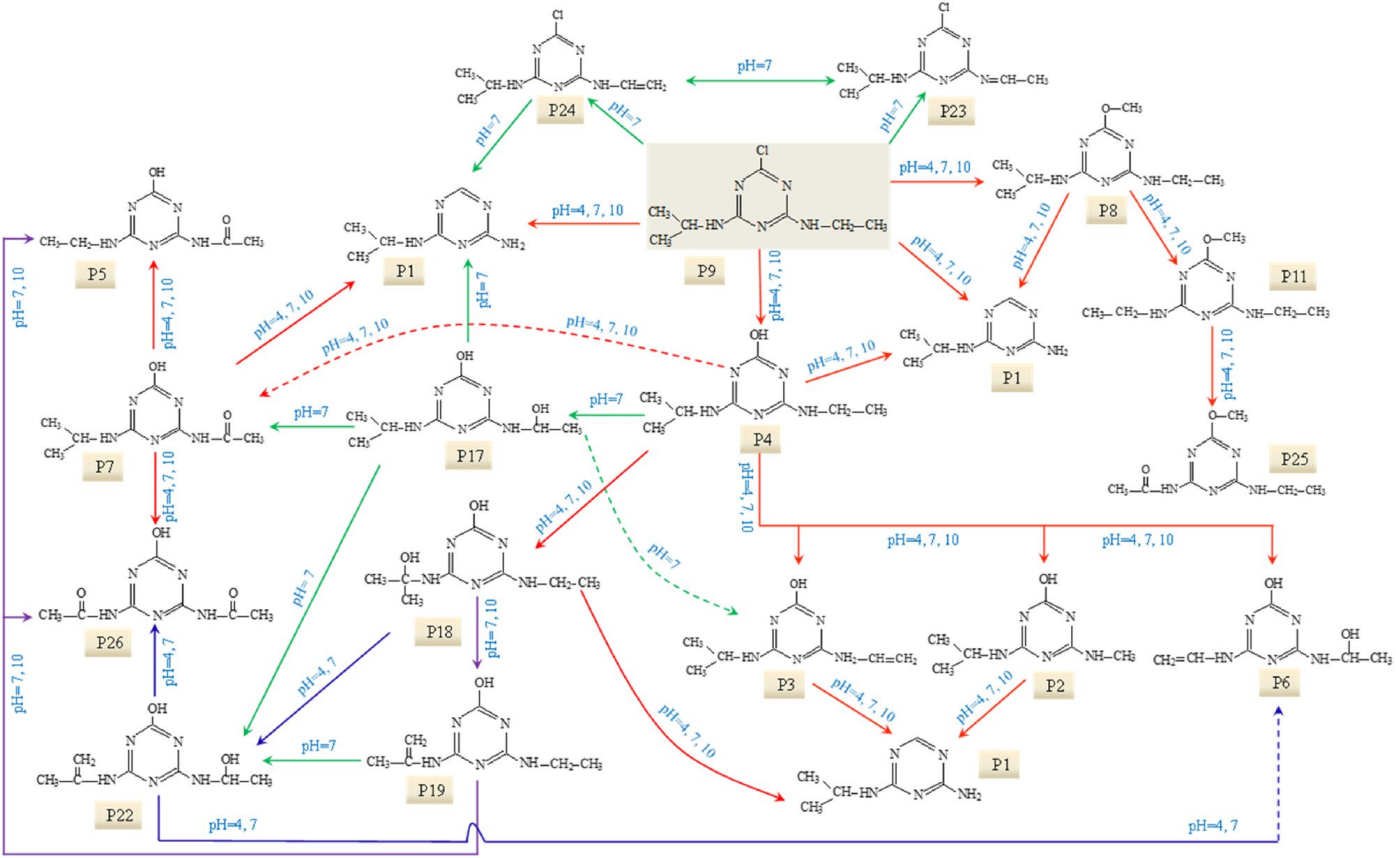
The proposed degradation pathway of ATZ in UV/TiO_2_ process.

According to previous studies and the MS spectra, the molecular structures of P22–P26 were deduced^29,31,35^, as shown in Supplementary Table S13. Compared to UV and UV/H_2_O_2_ process, five new photooxidation products of ATZ were identified during UV/TiO_2_ oxidation. UV/TiO_2_ treatment is an indirect heterogeneous photooxidation process, due to the addition of TiO_2_ nanoparticles^36^. Five steps occurred in heterogeneous system, as stated below: (1) ATZ molecular transferred from the bulk to TiO_2_ particle; (2) adsorption of ATZ onto the photon activated TiO_2_ surface; (3) photooxidation reaction; (4) desorption of oxidation products from TiO_2_ particles surface; (5) transfer of oxidation products from TiO_2_ surface to the bulk^37,38^. Photogenerated holes (*h*^+^_VB_) and ·OH were produced when TiO_2_ particles were irradiated by UV light. Furthermore, other free radicals (superoxide radical and hydroperoxide radical) were produced in UV/TiO_2_ system^39^, increased the types of oxidation reactions of ATZ. On the basis of identified photooxidation intermediates, the oxidation pathway of ATZ in UV/TiO_2_ process was proposed, as shown in Fig. 7.

### Chlorine demand in post-chlorination

**Figure 8:**
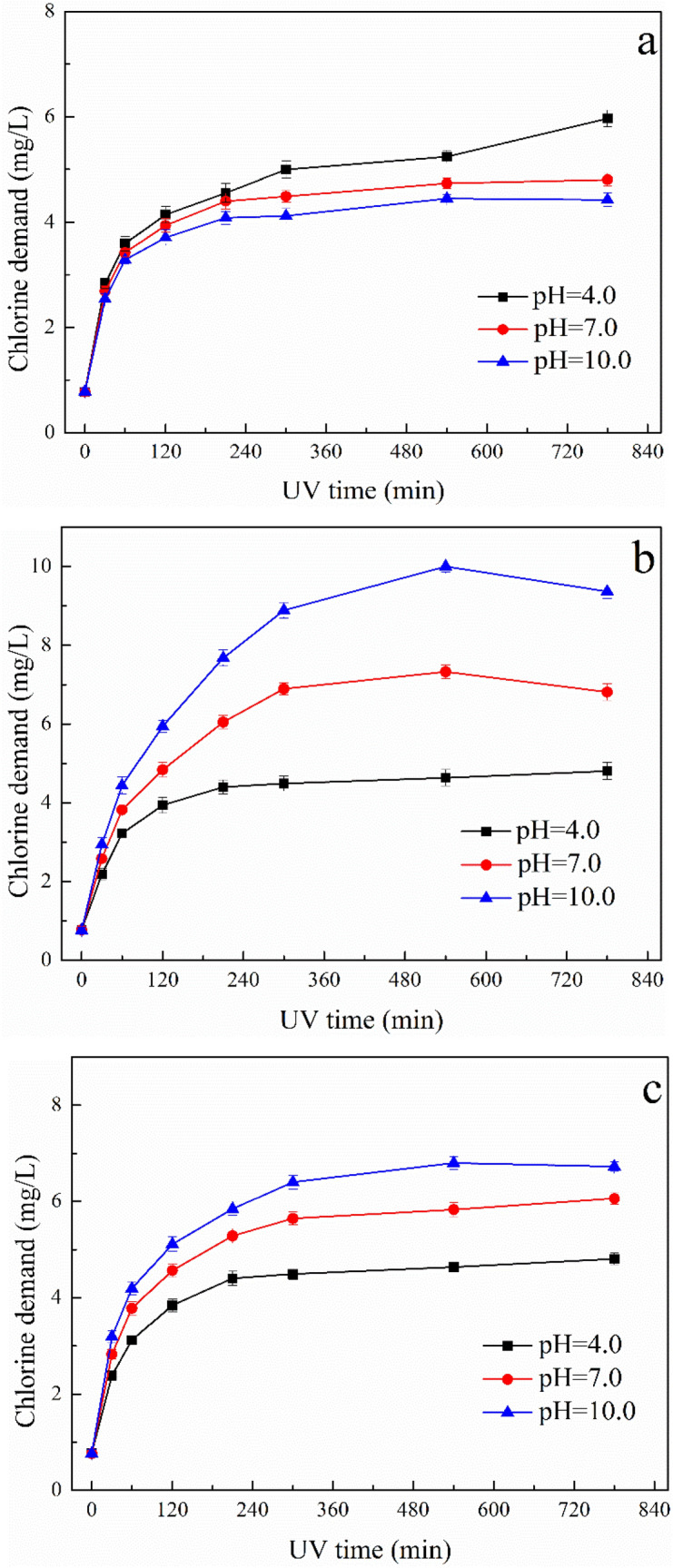
The chlorine demand of oxidized ATZ solutions after 24 h chlorination, (**a**) UV process; (**b**) UV/H_2_O_2_ process; **(c)** UV/TiO_2_ process.

Previous studies focus on DBPs have proved that chlorine demand is an important element related with the formation of DBPs during chlorination process^40,41^. The chlorine demand of ATZ solution after UV, UV/H_2_O_2_ and UV/TiO_2_ treatment under different pH values are shown in Fig. 8.

Generally, the amount of chlorine consumed in chlorination treatment process increased along with the increase of photooxidation time. The increase of chlorine demand indicated the production of various photooxidation intermediates aggrandized the reactivity of oxidized solution toward chlorine. In order to confirm the chlorine demand of original ATZ solution, controlled trials were conducted, obtaining chlorine demand of 0.776 ± 0.08 with solution pH value as 7.0. In UV process, the chlorine demand increased with UV fluence, as shown in Fig. 8a. After 300 min irradiation, the chlorine demand reached 4.997 ± 0.16, 4.491 ± 0.11 and 4.121 ± 0.14 mg/L with photooxidation pH value at 4.0, 7.0 and 10.0, respectively. Further increase of UV irradiation time to 780 min, the increasing tendency of chlorine demand became slow, except the photooxidation pH of 4.0. Although chlorination process consumed the most chlorine under photooxidation pH of 4.0, the amounts of chlorine consumed under different photooxidation pH values were close. In UV/H_2_O_2_ process, the amount of chlorine consumed in chlorination process firstly increased sharply with the increase of irradiation time from 0 to 540 min, as shown in Fig. 8b. After 540 min irradiation, the chlorine demand reached 4.636 ± 0.21, 7.325 ± 0.18 and 10.003 ± 0.15 mg/L with photooxidation pH value at 4.0, 7.0 and 10.0, respectively. Further increase of photooxidation time to 780 min, the consumed chlorine exhibited slight downward trend. The influence of solution pH during photooxidation on chlorine demand was remarkable, the amount of chlorine consumed was the highest under photooxidation pH of 10.0. In UV/TiO_2_ process, the chlorine demand increased rapidly with the increase of irradiation time from 0 to 300 min, as shown in Fig. 8c. After 300 min irradiation, the chlorine demand reached 4.491 ± 0.11, 5.645 ± 0.13 and 6.402 ± 0.14 mg/L with photooxidation pH value at 4.0, 7.0 and 10.0, respectively. Further increase of UV irradiation made unconspicuous impact on chlorine demand. The key parameter that had an important effect on the chlorine demand was the solution pH during photooxidation, the amount of chlorine consumed was the highest as photooxidation pH of 10.0 under different photooxidation time.

In comparison, the amount of chlorine consumed by ATZ solution after UV/H_2_O_2_ treatment was the highest than direct UV photolysis and UV/TiO_2_ oxidation, especially under photooxidation pH of 10.0 after prolonged irradiation. The increase of chlorine demand depended on the production of specific oxidation intermediates. As for UV/H_2_O_2_ oxidation under solution pH 10.0, seventeen oxidation intermediates were identified including P20 and P21 only detected in this condition.

### Formation of DBPs

Five kinds of DBPs, including DCAA, TCAA, TCP, TCM and CHP, were detected after 24 h chlorination (under solution pH of 7.0) of the UV, UV/H_2_O_2_ and UV/TiO_2_ oxidized ATZ solution. In order to evaluate the effects of irradiation time and photooxidation pH on the formation of DBPs, raw ATZ solutions were chlorinated under solution pH of 7.0. The concentrations of DCAA, TCAA, TCP and TCM reached 0.425 ± 0.015, 0.533 ± 0.01, 0.114 ± 0.01 and 0.678 ± 0.105 μg/L , respectively, after 24 h chlorination. Meanwhile, CHP was undetected in the samples.

#### Formation of DBPs after UV treatment

**Figure 9:**
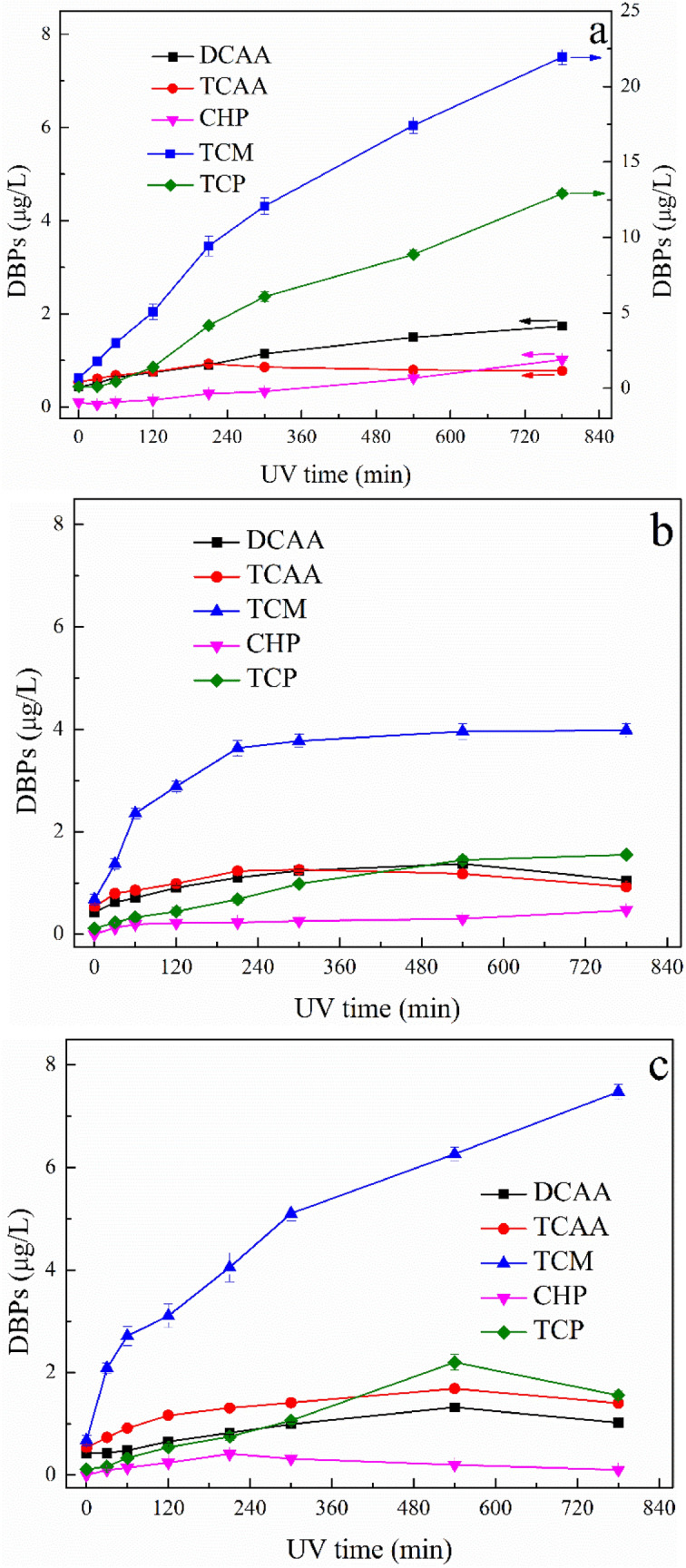
DBPs formations of UV oxidized ATZ solutions: (**a**) pH = 4.0; (**b**) pH = 7.0; **(c)** pH = 10.0.

As shown in Fig. 9, irradiation time and photooxidation pH made distinct influences on the formation of DBPs. Generally, TCM dominated the DBPs generation and direct photolysis significantly changed DBPs formation and speciation. The effect of irradiation time in UV photolysis system under solution pH of 4.0 on the formation of DBPs is depicted in Fig. 9a. The formation of TCM and TCP during chlorination process increased sharply with the increase of irradiation time. The concentration of DCAA and CHP increased slowly with prolonged UV oxidation. However, the concentration of TCAA increased slightly at the early stage of reaction time, then decreased as the irradiation time exceeded 210 min. The effects of irradiation time in UV photolysis system under solution pH of 7.0 and 10.0 on the formation of DBPs is depicted in Fig. 9b, c, respectively. The concentrations of TCM and TCP under photooxidation pH of 7.0 and 10.0 declined significantly compared to the output under pH 4.0. Under photooxidation pH of 7.0, the concentration of DBPs basically remains stable after 210 min irradiation, except TCP. However, under photooxidation pH of 10.0, the concentrations of CHP, TCP, DCAA and TCAA exhibited downward trend as the irradiation time increased.

It is worth noting that CHP was undetected during chlorination of raw ATZ solution. This phenomenon indicated that ATZ did not react with chlorine to produced CHP in the bulk. The variation tendency of DBPs observed might have been related to the oxidation of ATZ by direct UV photolysis and the production oxidation intermediates^42,43^. It has been reported that direct photolysis of organics could break drown the hydrophobic parts, forming hydrophilic and polar intermediates, and increased DBPs formation potentials^44,45^.

#### Formation of DBPs after UV/H_2_O_2_ treatment

**Figure 10:**
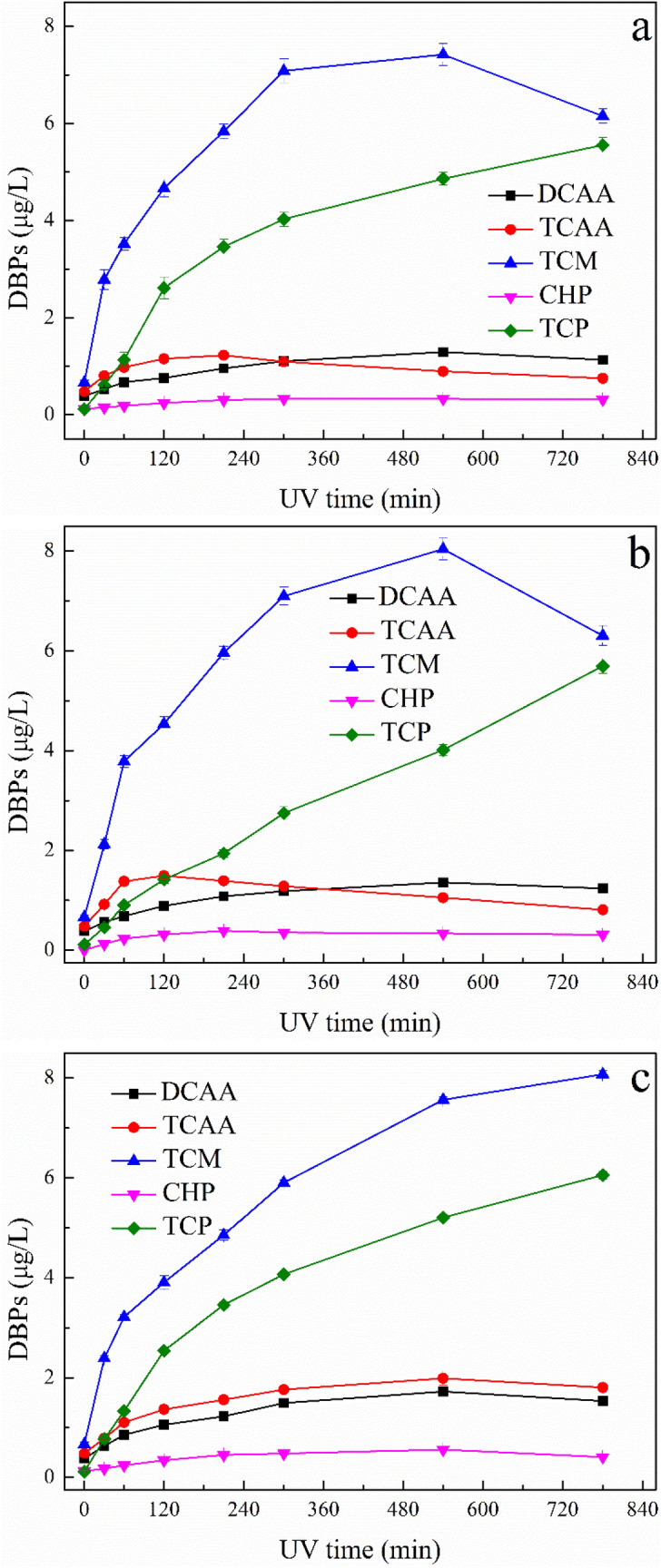
DBPs formations of UV/H_2_O_2_ oxidized ATZ solutions: (**a**) pH = 4.0; (**b**) pH = 7.0; **(c)** pH = 10.0.

The effects of irradiation time and UV/H_2_O_2_ oxidation pH on the formation of DBPs are depicted in Fig. 10. Compared to other DBPs, much more TCM and TCP were formed in UV/H_2_O_2_ treated ATZ solutions. Under photooxidation pH of 4.0, the TCM and TCP formations were greatly increased with increase of irradiation time at initial reaction stage, as shown in Fig. 10a. After 210 min oxidation in UV/H_2_O_2_ system, the concentration of TCAA began to decrease. Furthermore, the formation of TCM was suppressed after longer oxidation. Figure 10b shows the formations of DBPs in UV/H_2_O_2_ treated ATZ solutions under photooxidation pH of 7.0. The influence of the irradiation time on the formation of DBPs was also remarkable, for the destruction of ATZ during photooxidation process improved the reactivity of solution toward chloride. TCM concentration increased rapidly, reaching its maximum level after 540 min photooxidation. The formation of TCP increased with increase of irradiation time, on the contrary, the formations of TCAA and CHP were suppressed after longer oxidation. The formations of DBPs in UV/H_2_O_2_ treated ATZ solutions under photooxidation pH of 10.0 is shown in Fig. 10c. The formations of TCM and TCP were vastly promoted by UV/H_2_O_2_ oxidation at the entire stage of reaction time. However, the concentrations of TCAA, DCAA and CHP in photo-oxidized solutions only increased within early stage of reaction time.

At pH 4.0, fourteen oxidation intermediates were identified, furthermore, fifteen and seventeen intermediates were detected at pH 7.0 and 10.0, respectively. The variation tendency of DBPs in the UV/H_2_O_2_ oxidized ATZ solutions under different pH values during chlorination process might due to the diverse species and concentrations of ATZ intermediates.

#### Formation of DBPs after UV/TiO_2_ treatment

**Figure 11:**
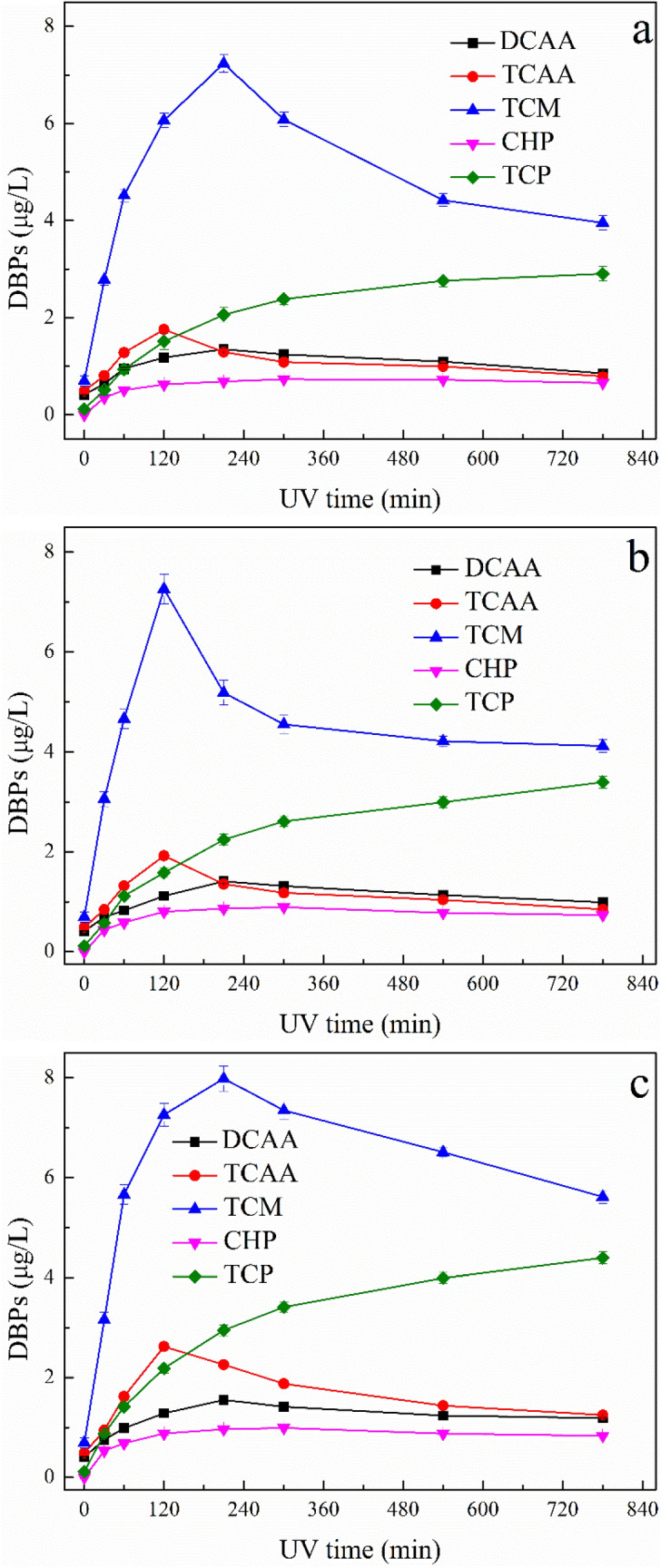
DBPs formations of UV/TiO_2_ oxidized ATZ solutions: (**a**) pH = 4.0; (**b**) pH = 7.0; **(c)** pH = 10.0.

The concentrations of DBPs formed under present experimental conditions are shown in Fig. 11. Without UV/ TiO_2_ oxidation, only small amounts of DBPs were formed. However, the formations of DBPs were vastly promoted in the UV/TiO_2_ treated ATZ solutions during 24 h chlorination, and TCM and TCP exhibited the highest concentrations compared to DCAA, TCAA and CHP. Figure 11a shows the formations of DBPs in UV/TiO_2_ treated ATZ solutions under photooxidation pH of 4.0. The formations of TCM and TCAA were greatly promoted at the early stage of reaction time, then decreased as the oxidation reaction continues. Figure 11b, c show the formations of DBPs in UV/TiO_2_ treated ATZ solutions under photooxidation pH of 7.0 and 10.0, respectively, and the formation trend of DBPs were similar to the case of pH 4.0. The similar tendency of DBPs in UV/TiO_2_ oxidized ATZ solutions under different pH values during chlorination process might due to the similar main intermediates of ATZ (P1–P8, P11, P18).

## Conclusion

This study investigated the effect of solution pH on the oxidation pathway of ATZ in UV, UV/H_2_O_2_ and UV/TiO_2_ oxidation system and the impact of photooxidation on the DBPs formation of ATZ solution during post-chlorination was followed. The structures of the main photooxidation intermediates were deduced on the basis of MS and MS/MS spectra, which showed that the photooxidation of ATZ in UV/H_2_O_2_ and UV/TiO_2_ system was significantly pH-dependent processes. The plausible degradation pathways of ATZ in photooxidation systems were proposed. The photooxidation pH and irradiation time had distinctly impact on the DBPs formation in oxidized ATZ solutions during post-chlorination. The formations of DBPs were enormously promoted in the early stage of photooxidation reaction time, TCM and TCP were the main DBPs formed. The increased DBPs concentrations in oxidized ATZ solutions might have been related to the combustion of ATZ and the production of oxidation intermediates. The observed phenomena in this study indicated that the DBPs formation in micropollutants containing water after pre-oxidation might be promoted. This tendency should be seriously evaluated and appropriately resolved when photooxidation processes are applied to water treatment.

## Supplementary information

Supplementary information.
