# Supplementary material for: Photooxidation of atrazine and its influence on disinfection byproducts formation during post-chlorination: effect of solution pH and mechanism
Source: Sci Rep. 2020 Nov 23;10:20355. doi: 10.1038/s41598-020-77006-0 (PMC7684306; doi:10.1038/s41598-020-77006-0)
Supplement: Supplementary file 1 — Supplementary information. [file 41598_2020_77006_MOESM1_ESM.doc]

*Supplementary Materials*

**Photooxidation of atrazine and its influence on disinfection byproducts formation** [**during post-chlorination**](https://www.sciencedirect.com/science/article/pii/S1385894719311209)**: effect of solution pH and mechanism**

Yucan Liu^a^, Kai Zhu^b^,*, Huayu Zhu^c^, Min Zhao^b^, Lihua Huang^b^, Bin Dong^b^ and Qianjin Liu^b^,*

^a^ School of Civil Engineering, Yantai University, Yantai, 264005, China

^b^ Shandong Provincial Key Laboratory of Water and Soil Conservation and Environmental Protection, College of resources and environment, Linyi University, Linyi, 276000, China

^c^ School of Chemistry & Chemical Engineering, Linyi University, Linyi 276000, China

*Corresponding authors at: Shandong Provincial Key Laboratory of Water and Soil Conservation and Environmental Protection, College of resources and environment, Linyi University, Linyi, 276000, China

E-mail addresses: [zhukai@lyu.edu.cn](mailto:zhukai@lyu.edu.cn) (K. Zhu), [liuqianjin@lyu.edu.cn](mailto:liuqianjin@lyu.edu.cn) (Q. Liu)

**Contents**

**Text S1** Parameters of UPLC and MS for identifying degradation products of ATZ 4

**Text S2** Detection method of Haloacetic acids 5

**Text S3** Detection method of volatile disinfection byproducts 6

**Table S1** Retention time (RT), cone voltage (CV), transition ions, collision energy (CE), limits of detection (LODs) and limits of quantification (LOQs) for nine HAAs. 7

**Table S2** Retention time (RT), electron ionization energy (EI), transition ions, collision energy (CE), limits of detection (LODs) and limits of quantification (LOQs) for volatile disinfection byproducts 8

**Table S3** Kinetic parameters of ATZ photo-oxidation at different solution pH condition in UV process. 9

**Table S4** Kinetic parameters of ATZ photo-oxidation at different solution pH condition in UV/H_2_O_2_ process. 10

**Table S5** Kinetic parameters of ATZ photo-oxidation at different solution pH condition in UV/TiO_2_ process. 11

**Table S6** Retention time (RT) and MS spectral information in full scan modes of ATZ and its intermediates. 12

**Table S7** Retention time (RT) and MS/MS spectral information in daughter scan modes of ATZ and its intermediates. 13

**Table S8** Precursor ions in daughter scan of ATZ and its intermediates; molecular weight (MW), formula, proposed structure and chemical name for the intermediates of ATZ during UV irradiation treatment. 14

**Table S9** Retention time (RT) and MS spectral information in full scan modes of ATZ and its intermediates. 15

**Table S10** Retention time (RT) and MS/MS spectral information in daughter scan modes of ATZ and its intermediates. 16

**Table S11** Precursor ions in daughter scan of ATZ and its intermediates; molecular weight (MW), formula, proposed structure and chemical name for the intermediates of ATZ in UV/H_2_O_2_ process. 17

**Table S12** MS fragment information for the new intermediates of ATZ in UV/TiO_2_ process. 18

**Table S13** Precursor ions in daughter scan of ATZ and its intermediates; molecular weight (MW), formula, proposed structure and chemical name for the intermediates of ATZ in UV/TiO_2_ process. 19

**Fig. S1** Effect of storage time on concentration of ATZ without UV irradiation: (a) dark control experiment; (b) 5 mg/L H_2_O_2_; (c) 5mg/L TiO_2_. 20

**Fig. S2** The fitting of different solution pH values for ATZ to a pseudo-first order kinetic model: (a) UV process; (b) UV/H_2_O_2_ process; (c) UV/TiO_2_ process. 21

**Fig. S3** Molecular structure and MS/MS spectrum of P1 (ESI^+^, CE=22 eV). 22

**Fig. S4** Molecular structure and MS/MS spectrum of P2 (ESI^+^, CE=22 eV). 23

**Fig. S5** Molecular structure and MS/MS spectrum of P3 (ESI^+^, CE=0 eV). 24

**Fig. S6** Molecular structure and MS/MS spectrum of P4 (ESI^+^ and ESI^-^, CE=23 eV). 25

**Fig. S7** Molecular structure and MS/MS spectrum of P5 (ESI^+^ and ESI^-^, CE=16 eV). 26

**Fig. S8** Molecular structure and MS/MS spectrum of P6 (ESI^+^ and ESI^-^, CE=24 eV). 27

**Fig. S9** Molecular structure and MS/MS spectrum of P7 (ESI^+^, CE=26 eV). 28

**Fig. S10** Molecular structure and MS/MS spectrum of P8 (ESI^+^, CE=22 eV). 29

**Fig. S11** Molecular structure and MS/MS spectrum of P9 (ESI^+^, CE=22 eV). 30

**Fig. S12** Molecular structure and MS/MS spectrum of P10 (ESI^+^, CE=12 eV). 31

**Fig. S13** Molecular structure and MS/MS spectrum of P12 (ESI^+^, CE=20 eV). 32

**Fig. S14** Molecular structure and MS/MS spectrum of P13 (ESI^+^, CE=15 eV). 33

**Fig. S15** Molecular structure and MS/MS spectrum of P14 (ESI^+^ and ESI^-^, CE=20 eV). 34

**Fig. S16** Molecular structure and MS/MS spectrum of P15 (ESI^+^ and ESI^-^, CE=20 eV). 35

**Fig. S17** Molecular structure and MS/MS spectrum of P16 (ESI^+^, CE=15 eV). 36

**Fig. S18** Molecular structure and MS/MS spectrum of P17 (ESI^+^, CE=18 eV). 37

**Fig. S19** Molecular structure and MS/MS spectrum of P18 (ESI^+^, CE=17 eV). 38

**Fig. S20** Molecular structure and MS/MS spectrum of P19 (ESI^+^, CE=15 eV). 39

**Fig. S21** Molecular structure and MS/MS spectrum of P20 (ESI^+^ and ESI^-^, CE=20 eV). 40

**Fig. S22** Molecular structure and MS/MS spectrum of P21 (ESI^+^ and ESI^-^, CE=20 eV). 41

**Text S1** Parameters of UPLC and MS for identifying degradation products of ATZ

(1) Instruments: Ultra-performance liquid chromatography-electrospray tandem mass spectrometry (UPLC-ESI-MS/MS, Waters, Milford, MA, USA).

(2) Separation column: ACQUITY™ UPLC BEH C_8_ column (2.1 mm×100 mm, 1.7 μm particle, Waters).

(3) Mobile phases: Mobile phase A-methanol, mobile phase B-ultrapure water.

(4) Flow rate: 0.2 mL/min.

(5) Column temperature: 35 ºC.

(6) Elution gradient: The elution started with 10% A for 3 minutes, the concentration of A was increased to 70% within 18 minutes, and then the concentration of A was increased to 100% within 22 minutes and retained for 3 minutes, finally it was dropped back to 10% and run for 3 minutes for equilibrium before the next injection. A total acquisition time of one run analysis was 28 min.

(7) Injection volume: 10 μL.

(8) Mass spectrometry condition: The source temperature and desolvation temperature were 110 ºC and 350 ºC, the capillary voltage was 3.3 kV, and the cone voltage was 35 V. Desolvation gas (nitrogen gas) and cone gas (nitrogen gas) flows were set at 500 L/h and 30 L/h, respectively. Full scan data were acquired from *m*/*z* 50 to 300 at an acquisition rate of 0.2 sec/scan in both positive electrospray ionization (ESI^+^) mode and negative electrospray ionization (ESI^-^) mode. In order to obtain further information for analyzing the structure of intermediates, collision induced dissociation (CID) experiments in daughter scan were also conducted. Argon was used as collision gas in daughter scan model and its flow rate was at 0.12 mL/min. The collision energy for each product was optimized in the range from 15 to 35 eV.

**Text S2** Detection method of Haloacetic acids

(1) Instruments: Ultra-performance liquid chromatography-electrospray tandem mass spectrometry (UPLC-ESI-MS/MS, Waters, Milford, MA, USA).

(2) Separation column: ACQUITY^TM^ UPLC HSS T3 separation column (2.1 mm×100 mm, 1.8 μm particle size, Waters).

(3) Mobile phases: Mobile phase A-methanol, Mobile phase B-ultrapure water with 0.0005% formic acid.

(4) Flow rate: 0.2 mL/min.

(5) Column temperature: 35 ºC.

(6) Elution gradient: The separation started with 1% A for 1.5 minutes, then it increased to 40% within 4 minutes and maintained for 3 minutes, and then A was increased to 90% and maintained for 2 minutes. Finally it was reduced back to 1% and maintained 3 minutes for equilibrium before the next injection.

(7) Injection volume: 10 μL.

(8) Mass spectrometry condition: Negative electrospray ionization (ESI^-^) mode. The source temperature and desolvation temperature were 110 ºC and 350 ºC, and the capillary voltage was 2.50 kV. Desolvation gas (nitrogen gas), cone gas (nitrogen gas) and collision gas (argon) flows were set at 350 L/h, 50 L/h and 0.12 mL/min, respectively.

Retention time (RT), cone voltage (CV), transition ions, collision energy (CE), limits of detection (LODs) and limits of quantification (LOQs) for nine haloacetic acids were shown in Table S1.

**Text S3** Detection method of volatile disinfection byproducts

(1) Instruments: A gas chromatograph (7890A) coupled with a triple quadrupole mass spectrometer (7000B) (Agilent Technologies, Palo Alto, CA, USA).

(2) Separation column: Agilent J&W HP-5MS fused silica capillary column (30m × 0.25 mm I.D. × 0.25 µm film thickness, (5%) phenyl-(95%) methylpolysiloxane).

(3) Chromatographic conditions: The carrier gas (helium) flow rate was 1.5 mL/min and the transfer line pressure was 1.5 psi. The chromatographic oven temperature was first held isothermally at 35 ºC for 3.6 min and then ramped up to 210 ºC at a rate of 35 ºC/min, where it was held for 1.4 min. The injector port temperature was 180 ºC and the transfer line was maintained at 280 ºC.

(4) Injection volume: 1 μL in the splitless mode.

(5) Mass spectrometry condition: The mass spectrometer was operated in the electron impact (EI) ionization mode. Nitrogen gas was used as a collision gas at a constant flow rate of 1.5mL/min. 2.25 mL/min of helium gas was used as the quenching gas. The ionization source and quadrupole were maintained at 230 ºC and 150 ºC, respectively. A solvent delay of 2.5 minutes was set to prevent detector overload and ionization source contamination. The ionization source filament emission current was set at 35 mA.

Retention time (RT), electron ionization energy (EI), transition ions, collision energy (CE), limits of detection (LODs) and limits of quantification (LOQs) for 13 volatile disinfection byproducts were shown in Table S2.

Table S1 Retention time (RT), cone voltage (CV), transition ions, collision energy (CE), limits of detection (LODs) and limits of quantification (LOQs) for nine HAAs.

| CAS  Registry Number | HAA | Retention time (min) | Cone Voltage  (V) | Transition ions | | | LODs^b^  (μg/L) | LOQs^c^  (μg/L) |
| --- | --- | --- | --- | --- | --- | --- | --- | --- |
|  |  |  |  | Precursor ion (*m/z*) | Product ion (*m/z*) | CE^a^ (eV) |  |  |
| 79-11-8 | MCAA | 2.61 | 20 | 92.87[CH_2_^35^ClCOO]^-^ | 34.8[^35^Cl]^-^ | 7 | 0.079 | 0.238 |
| 79-08-3 | MBAA | 3.20 | 15 | 136.83[CH_2_^79^BrCOO]^-^ | 78.8[^79^Br]^-^ | 11 | 0.022 | 0.067 |
| 79-43-6 | DCAA | 3.71 | 20 | 126.80 [CH^35^Cl_2_COO]^-^ | 82.9[CH^35^Cl_2_]^-^ | 11 | 0.014 | 0.042 |
| 5589-96-8 | BCAA | 3.95 | 20 | 172.80 [CH^35^Cl^81^BrCOO]^-^ | 128.8[CH^35^Cl^81^Br]^-^ | 11 | 0.016 | 0.049 |
| 631-64-1 | DBAA | 4.14 | 20 | 216.76[CH^79^Br^81^BrCOO]^-^ | 172.8[CH^79^Br^81^Br]^-^ | 9 | 0.024 | 0.072 |
| 76-03-9 | TCAA | 4.80 | 20 | 160.79 [C^35^Cl_2_^37^ClCOO]^-^ | 116.8[C^35^Cl_2_^37^Cl]^-^ | 7 | 0.018 | 0.054 |
| 71133-14-7 | BDCAA | 4.98 | 20 | 206.76[C^35^Cl_2_^79^BrCOO]^-^ | 162.8[C^35^Cl_2_^79^Br]^-^ | 7 | 0.012 | 0.034 |
| 5278-95-5 | CDBAA | 5.23 | 20 | 250.74 [C^35^Cl^79^Br_2_COO]^-^ | 206.8[C^35^Cl^79^Br_2_]^-^ | 7 | 0.017 | 0.05 |
| 75-96-7 | TBAA | 5.51 | 30 | 250.74[C^79^Br_2_^81^Br]^-^ | 78.8[^79^Br]^-^ | 17 | 0.018 | 0.055 |

^a^Collision energy; ^b^Limits of detection; ^c^Limits of quantification.

Table S2 Retention time (RT), electron ionization energy (EI), transition ions, collision energy (CE), limits of detection (LODs) and limits of quantification (LOQs) for volatile disinfection byproducts

| CAS Registry Number | Analyst | RT^a^  (min) | DT^b^  (ms) | EI^c^  (eV) | Quantitative transition | | |  | Qualitative transition | | | LODs^e^  (μg/L) | LOQs^f^  (μg/L) |
| --- | --- | --- | --- | --- | --- | --- | --- | --- | --- | --- | --- | --- | --- |
|  |  |  |  |  | Precursor ion (*m/z*) | Product ion (*m/z*) | CE^d^ |  | Precursor ion (*m/z*) | Product ion (*m/z*) | CE^d^ |  |  |
| 67-66-3 | TCM | 2.61 | 240 | 50 | 82.9[CH^35^Cl_2_]^+^ | 47.0[C^35^Cl]^+^ | 27 |  | 117.9[CH^35^Cl_3_]^+^ | 82.9[CH^35^Cl_2_]^+^ | 31 | 0.004 | 0.012 |
| 545-06-2 | TCAN | 3.20 | 120 | 55 | 107.9[C^35^Cl_2_CN]^+^ | 72.9[C^35^ClCN]^+^ | 35 |  | 81.9[C^35^Cl_2_]^+^ | 47.0[C^35^Cl]^+^ | 27 | 0.003 | 0.010 |
| 107-14-2 | MCAN | 3.27 | 120 | 55 | 74.9[CH_2_^35^ClCN]^+^ | 48.0[CH^35^Cl]^+^ | 4 |  | 77.0[CH_2_^37^ClCN]^+^ | 50.0[CH^37^Cl]^+^ | 4 | 0.006 | 0.017 |
| 75-27-4 | BDCM | 3.71 | 240 | 50 | 82.9[CH^35^Cl_2_]^+^ | 47.0[C^35^Cl]^+^ | 27 |  | 128.6[CH^35^Cl^81^Br]^+^ | 48.0CH^35^Cl]^+^ | 45 | 0.006 | 0.019 |
| 3018-12-0 | DCAN | 3.95 | 120 | 50 | 73.9[CH^35^ClCN]^+^ | 47.0[C^35^Cl]^+^ | 20 |  | 81.9[C^35^Cl_2_]^+^ | 46.9[C^35^Cl]^+^ | 27 | 0.004 | 0.012 |
| 513-88-2 | 1,1-DCP | 4.14 | 120 | 50 | 91.1[CH^35^ClCOCH_3_]^+^ | 62.9[CH^35^ClCH_3_]^+^ | 3 |  | 82.9[CH^35^Cl_2_]^+^ | 46.9[C^35^Cl]^+^ | 27 | 0.010 | 0.029 |
| 590-17-0 | MBAN | 4.74 | 80 | 55 | 120.9[CH_2_^81^BrCN]^+^ | 40.1[CH_2_CN]^+^ | 10 |  | 118.9[CH_2_^79^BrCN]^+^ | 40.0[CH_2_CN]^+^ | 10 | 0.014 | 0.045 |
| 76-06-2 | CHP | 4.80 | 80 | 55 | 116.9[C^35^Cl_3_]^+^ | 81.9[C^35^Cl_2_]^+^ | 35 |  | 81.9[C^35^Cl_2_]^+^ | 46.9[C^35^Cl]^+^ | 27 | 0.007 | 0.022 |
| 124-48-1 | DBCM | 4.98 | 80 | 55 | 128.9[CH^35^Cl^79^Br]^+^ | 48.0[CH^35^Cl]^+^ | 45 |  | 208.0[CH^35^Cl^81^Br^79^Br]^+^ | 129.0[CH^35^Cl^81^Br]^+^ | 32 | 0.008 | 0.026 |
| 83463-62-1 | BCAN | 5.23 | 240 | 70 | 73.9[CH^35^ClCN]^+^ | 47.0[C^35^Cl]^+^ | 20 |  | 154.9[CH^35^Cl^81^BrCN]^+^ | 73.9[CH^35^ClCN]^+^ | 8 | 0.009 | 0.028 |
| 918-00-3 | 1,1,1-TCP | 5.51 | 120 | 55 | 124.8[C^35^Cl_2_COCH_3_]^+^ | 97.0[C^35^Cl_2_CH_3_]^+^ | 3 |  | 97.0[C^35^Cl_2_CH_3_]^+^ | 61.0[C^35^ClCH_2_]^+^ | 27 | 0.008 | 0.024 |
| 78-75-1 | IS | 5.62 | 120 | 55 | 120.9[CH_2_CH^79^BrCH_3_]^+^ | 41.1[CH_2_CHCH_2_]^+^ | 9 |  | 123.0[CH_2_CH^81^BrCH_3_]^+^ | 41.1[CH_2_CHCH_2_]^+^ | 9 | 0.011 | 0.035 |
| 75-25-2 | TBM | 5.91 | 120 | 65 | 172.9[CH^81^Br^79^Br]^+^ | 93.9[CH^81^Br]^+^ | 43 |  | 251.8[CH^81^Br^79^Br_2_]^+^ | 172.7[CH^81^Br^79^Br]^+^ | 38 | 0.012 | 0.040 |
| 3252-43-5 | DBAN | 6.15 | 120 | 65 | 117.9[CH^79^BrCN]^+^ | 90.9[C^79^Br]^+^ | 20 |  | 199.0[CH^79^Br^81^Br CN]^+^ | 117.9[CH^79^Br CN]^+^ | 8 | 0.004 | 0.012 |

^a^Retention time; ^b^Dwell time; ^c^Electron ionization energy; ^d^Collision energy; ^e^Limits of detection; ^f^Limits of quantification.

**Table S3** Kinetic parameters of ATZ photo-oxidation at different solution pH condition in UV process.

| pH | 4 | 7 | 10 |
| --- | --- | --- | --- |
| *k* (min^-1^) | 0.00779 | 0.01545 | 0.01308 |
| *R*^2^ | 0.9943 | 0.9984 | 0.9960 |

**Table S4** Kinetic parameters of ATZ photo-oxidation at different solution pH condition in UV/H_2_O_2_ process.

| pH | 4 | 7 | 10 |
| --- | --- | --- | --- |
| *k* (min^-1^) | 0.00842 | 0.01703 | 0.01253 |
| *R*^2^ | 0.9940 | 0.9977 | 0.9975 |

**Table S5** Kinetic parameters of ATZ photo-oxidation at different solution pH condition in UV/TiO_2_ process.

| pH | 4 | 7 | 10 |
| --- | --- | --- | --- |
| *k* (min^-1^) | 0.02042 | 0.01765 | 0.01289 |
| *R*^2^ | 0.9988 | 0.9968 | 0.9960 |

**Table S6** Retention time (RT) and MS spectral information in full scan modes of ATZ and its intermediates.

| Name | RT  (min) | MS spectral  (ESI^+^) | MS spectral  (ESI^-^) |
| --- | --- | --- | --- |
| P1 | 7.42 |  |  |
| P2 | 9.89 |  |  |
| P3 | 11.90 |  |  |
| P4 | 12.60 |  |  |
| P5 | 14.06 |  |  |
| P6 | 14.46 |  |  |
| P7 | 14.88 |  |  |
| P8 | 15.20 |  |  |
| P9 | 15.44 |  |  |
| P10 | 5.02 |  |  |
| P11 | 7.54 |  |  |

**Table S7** Retention time (RT) and MS/MS spectral information in daughter scan modes of ATZ and its intermediates.

| Name | RT  (min) | MS spectral  (ESI^+^) | MS spectral  (ESI^-^) |
| --- | --- | --- | --- |
| P1 | 7.42 |  |  |
| P2 | 9.89 |  |  |
| P3 | 11.90 |  |  |
| P4 | 12.60 |  |  |
| P5 | 14.06 |  |  |
| P6 | 14.46 |  |  |
| P7 | 14.88 |  |  |
| P8 | 15.20 |  |  |
| P9 | 15.44 |  |  |
| P10 | 5.02 |  |  |
| P11 | 7.54 |  |  |

**Table S8** Precursor ions in daughter scan of ATZ and its intermediates; molecular weight (MW), formula, proposed structure and chemical name for the intermediates of ATZ during UV irradiation treatment.

| Name | ESI Model | MS Fragment ions  (*m/z*) | Precursor ions  (*m/z*) | MW  (Da) | Formula | Proposed structure | Chemical name |
| --- | --- | --- | --- | --- | --- | --- | --- |
| P1 | ESI^+^ | 112, 85, 70, 68 | 154 | 153 | C_6_H_11_N_5_ |  | 4-Isopropylamino-6-amino-*s*-triazine |
| P2 | ESI^+^ | 156, 142, 114, 97, 71, 69 | 184 | 183 | C_7_H_13_N_5_O |  | 2-Methoxy-4-methylamino-6-isopropylamino-*s*-triazine |
| P3 | ESI^+^ | 145, 97, 89, 71, 65 | 196 | 196 | C_8_H_14_N_5_O |  | 2-Hydroxy-4-isopropylamino-6-vinylamino-*s*-triazine |
| P4 | ESI^+^ | 156, 128, 114, 97, 86, 69 | 198 | 197 | C_8_H_15_N_5_O |  | 2-Hydroxy-4-ethylamino-6-isopropylamines-*s*-triazine |
|  | ESI^-^ | 168, 154, 125, 111, 83, 69 | 196 |  |  |  |  |
| P5 | ESI^+^ | 156, 139, 113, 96, 85, 71 | 198 | 197 | C_7_H_11_N_5_O_2_ |  | 2-Hydroxy-4-acetamido-6-ethylamino-*s*-triazine |
|  | ESI^-^ | 137, 111, 83, 69 | 196 |  |  |  |  |
| P6 | ESI^+^ | 156, 153, 127, 113, 85, 71 | 198 | 197 | C_7_H_11_N_5_O_2_ |  | 2-Hydroxy-4-(2-hydroxy-ethylamino)-6-vinylamino-*s*-triazine |
|  | ESI^-^ | 151, 125, 111, 83 | 196 |  |  |  |  |
| P7 | ESI^+^ | 170, 128, 86, 68 | 212 | 211 | C_8_H_13_N_5_O_2_ |  | 2-Hydroxy-4-acetamido-6-isopropylamino-*s*-triazine |
| P8 | ESI^+^ | 182, 170, 142, 128, 114, 97 | 212 | 211 | C_9_H_17_N_5_O |  | 2-Methoxy-4-isopropylamino-6-ethylamino-*s*-triazine |
| P9 | ESI^+^ | 174, 146, 96, 71, 68 | 216 | 215.7 | C_8_H_14_N_5_Cl |  | 2-Chloro-4-ethylamino-6-isopropylamino-*s*-triazine |
| P10 | ESI^+^ | 81, 72 | 139 | 138 | C_5_H_6_N_4_O |  | 2-Hydroxy-4-vinylamino-*s*-triazine |
| P11 | ESI^+^ | 198, 220 | 198 | 197 | C_8_H_15_N_5_O |  | 2-Methoxy-4,6-diethylamino-*s*-triazine |
|  | ESI^-^ | 196 | 196 |  |  |  |  |

**Table S9** Retention time (RT) and MS spectral information in full scan modes of ATZ and its intermediates.

| Name | RT  (min) | MS spectral  (ESI^+^) | MS spectral  (ESI^-^) |
| --- | --- | --- | --- |
| P12 | 6.70 |  |  |
| P13 | 8.58 |  |  |
| P14 | 11.38 |  |  |
| P15 | 11.64 |  |  |
| P16 | 16.60 |  |  |
| P17 | 3.92 |  |  |
| P18 | 7.90 |  |  |
| P19 | 9.20 |  |  |
| P20 | 9.77 |  |  |
| P21 | 9.95 |  |  |

**Table S10** Retention time (RT) and MS/MS spectral information in daughter scan modes of ATZ and its intermediates.

| Name | RT  (min) | MS spectral  (ESI^+^) | MS spectral  (ESI^-^) |
| --- | --- | --- | --- |
| P12 | 6.70 |  |  |
| P13 | 8.58 |  |  |
| P14 | 11.38 |  |  |
| P15 | 11.64 |  |  |
| P16 | 16.60 |  |  |
| P17 | 3.92 |  |  |
| P18 | 7.90 |  |  |
| P19 | 9.20 |  |  |
| P20 | 9.77 |  |  |
| P21 | 9.95 |  |  |

**Table S11** Precursor ions in daughter scan of ATZ and its intermediates; molecular weight (MW), formula, proposed structure and chemical name for the intermediates of ATZ in UV/H_2_O_2_ process.

| Name | ESI Model | MS Fragment ions (*m/z*) | Precursor ions  (*m/z*) | MW  (Da) | Formula | Proposed structure | Chemical name |
| --- | --- | --- | --- | --- | --- | --- | --- |
| P12 | ESI^+^ | 168, 152, 123, 115 | 210 | 209 | C_8_H_11_N_5_O |  | 2-Hydroxy-4-acetamido-6-isopropenylenylamino-*s*-triazine |
| P13 | ESI^+^ | 97, 139 | 139 | 138 | C_5_H_6_N_4_O |  | 4-acetamido-*s*-triazine |
| P14 | ESI^+^ | 184, 170, 142, 100, 85, 68 | 212 | 211 | C_8_H_13_N_5_O_2_ |  | 2-Hydroxy-4-acetamido-6-(2-hydroxyisopropylamino) -*s*-triazine |
|  | ESI^-^ | 182, 166, 136, 115, 111, 98 | 210 |  |  |  |  |
| P15 | ESI^+^ | 170, 103, 86 | 212 | 211 | C_8_H_13_N_5_O_2_ |  | 2-Hydroxy-4-ethylimine-6-(2-hydroxyisopropylamino) -*s*-triazine |
|  | ESI^-^ | 167, 152, 123, 115, 101, 66 | 210 |  |  |  |  |
| P16 | ESI^+^ | 173, 214 | 214 | 213 | C_7_H_8_N_5_OCl |  | 2-Chloro-4-vinylamino-6-acetamido-*s*-triazine |
| P17 | ESI^+^ | 196, 170, 143, 129 | 214 | 213 | C_8_H_15_N_5_O_2_ |  | 2-Hydroxy-4-(2-hydroxy-ethylamino)-6-isopropylamino-*s*-triazine |
| P18 | ESI^+^ | 214 | 214 | 213 | C_8_H_15_N_5_O_2_ |  | 2-Hydroxy-4-ethylamino-6-(2-hydroxyisopropylamino) -*s*-triazine |
| P19 | ESI^+^ | 218, 154, 127 | 196 | 195 | C_8_H_13_N_5_O |  | 2-Hydroxy-4-ethylamino-6-isopropenylenylamino-*s*-triazine |
| P20 | ESI^+^ | 172, 194 | 172 | 171 | C_5_H_9_N_5_O_2_ |  | 2-Hydroxy-4-(2-hydroxy-ethylamino)-6-amino-*s*-triazine |
|  | ESI^-^ | 170 | 170 |  |  |  |  |
| P21 | ESI^+^ | 172, 194 | 172 | 171 | C_5_H_6_N_5_Cl |  | 2-Chloro-4-vinylamino-6-amino-*s*-triazine |
|  | ESI^-^ | 170 | 170 |  |  |  |  |

**Table S12** MS fragment information for the new intermediates of ATZ in UV/TiO_2_ process.

| Name | RT  (min) | MS  (ESI^+^，*m/z*) | MS  (ESI^-^，*m/z*) |
| --- | --- | --- | --- |
| P22 | 6.96 | 212 (M+H), 234 (M+Na) | 210 (M-H) |
| P23 | 12.89 | 214 (M+H), 198 |  |
| P24 | 13.13 | 214 (M+H), 198 |  |
| P25 | 15.76 | 212 (M+H), 234 (M+Na) |  |
| P26 | 16.15 | 212 (M+H), 234 (M+Na) | 210 (M-H) |

**Table S13** Precursor ions in daughter scan of ATZ and its intermediates; molecular weight (MW), formula, proposed structure and chemical name for the intermediates of ATZ in UV/TiO_2_ process.

| Name | RT  (min) | MW (Da) | Formula | Proposed structure | Chemical name |
| --- | --- | --- | --- | --- | --- |
| P22 | 6.96 | 211 | C_8_H_13_N_5_O_2_ |  | 2-Hydroxy-4-(2-hydroxy-ethylamino)-6-isopropenylenylamino-*s*-triazine |
| P23 | 12.89 | 213 | C_8_H_12_N_5_Cl |  | 2-Chloro-4-ethylimine-6-isopropylamino-*s*-triazine |
| P24 | 13.13 | 213 | C_8_H_12_N_5_Cl |  | 2-Chloro-4-vinylamino-6-isopropylamino-*s*-triazine |
| P25 | 15.76 | 211 | C_8_H_13_N_5_O_2_ |  | 2-Methoxy-4-acetamindo-6-ethylamino-*s*-triazine |
| P26 | 16.15 | 211 | C_7_H_9_N_5_O_3_ |  | 2-Hydroxy-4,6-diacetamindo-*s*-triazine |











**Fig. S1** Effect of storage time on concentration of ATZ without UV irradiation: (a) dark control experiment; (b) 5 mg/L H_2_O_2_; (c) 5mg/L TiO_2_.


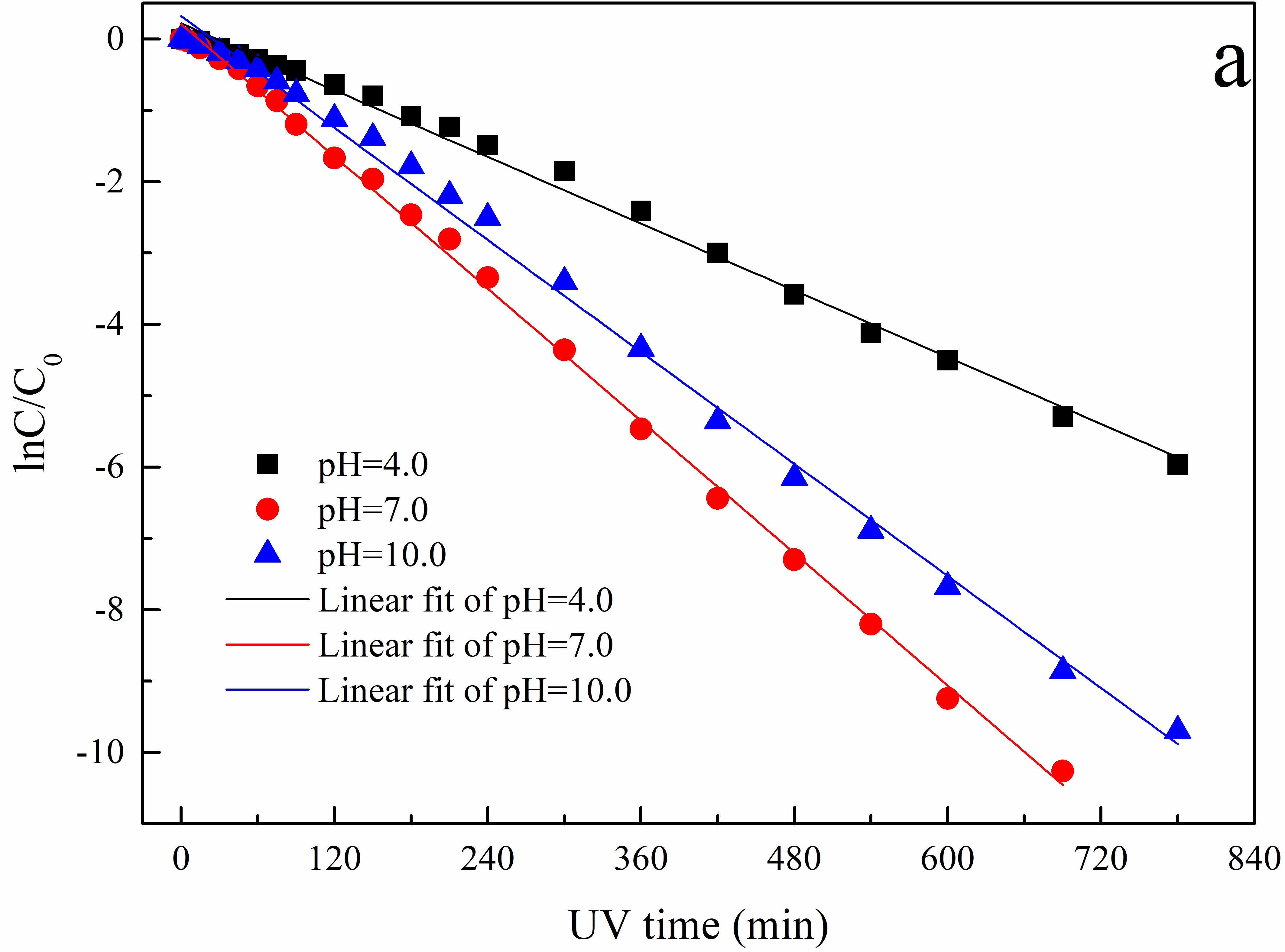


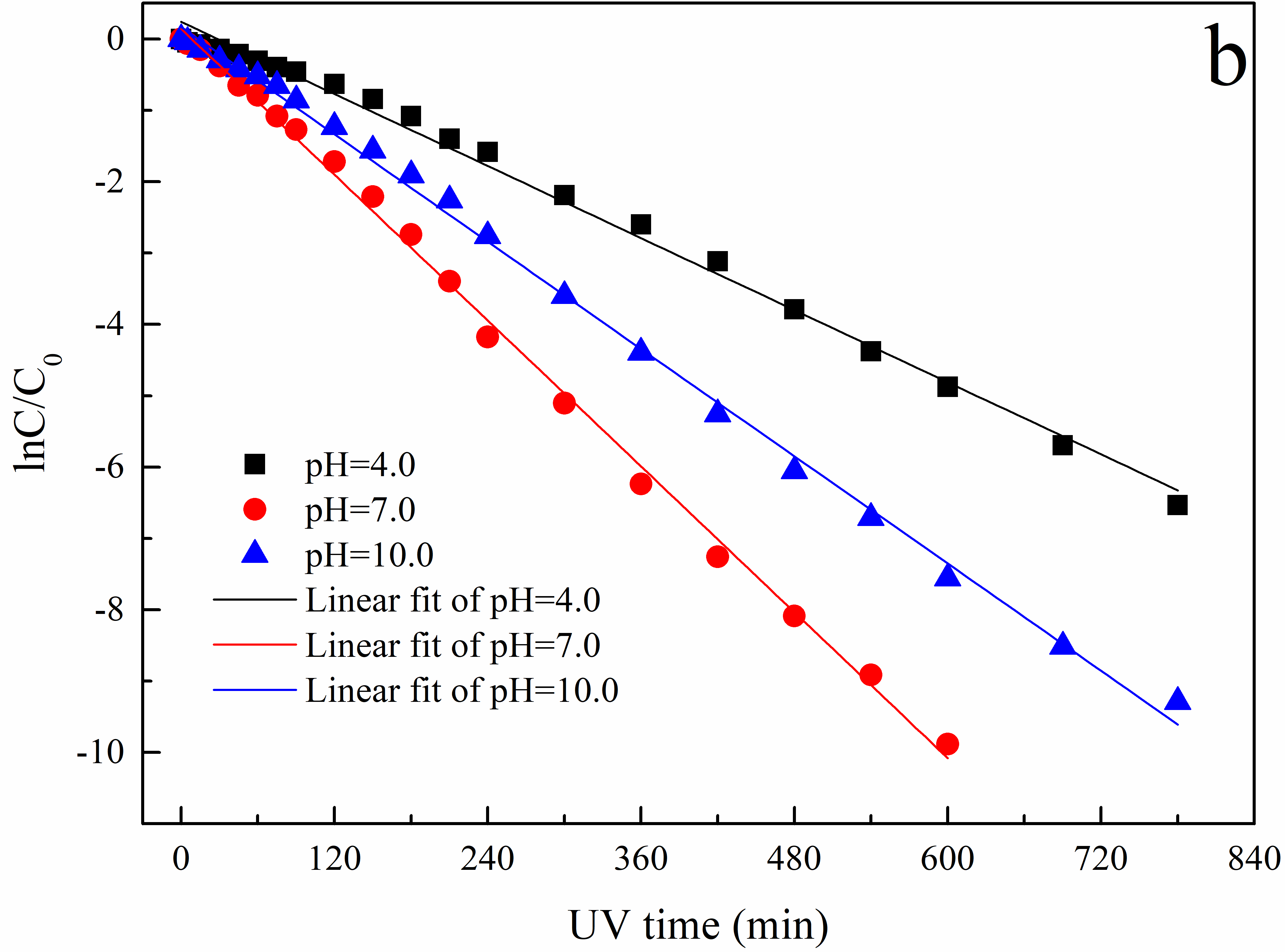


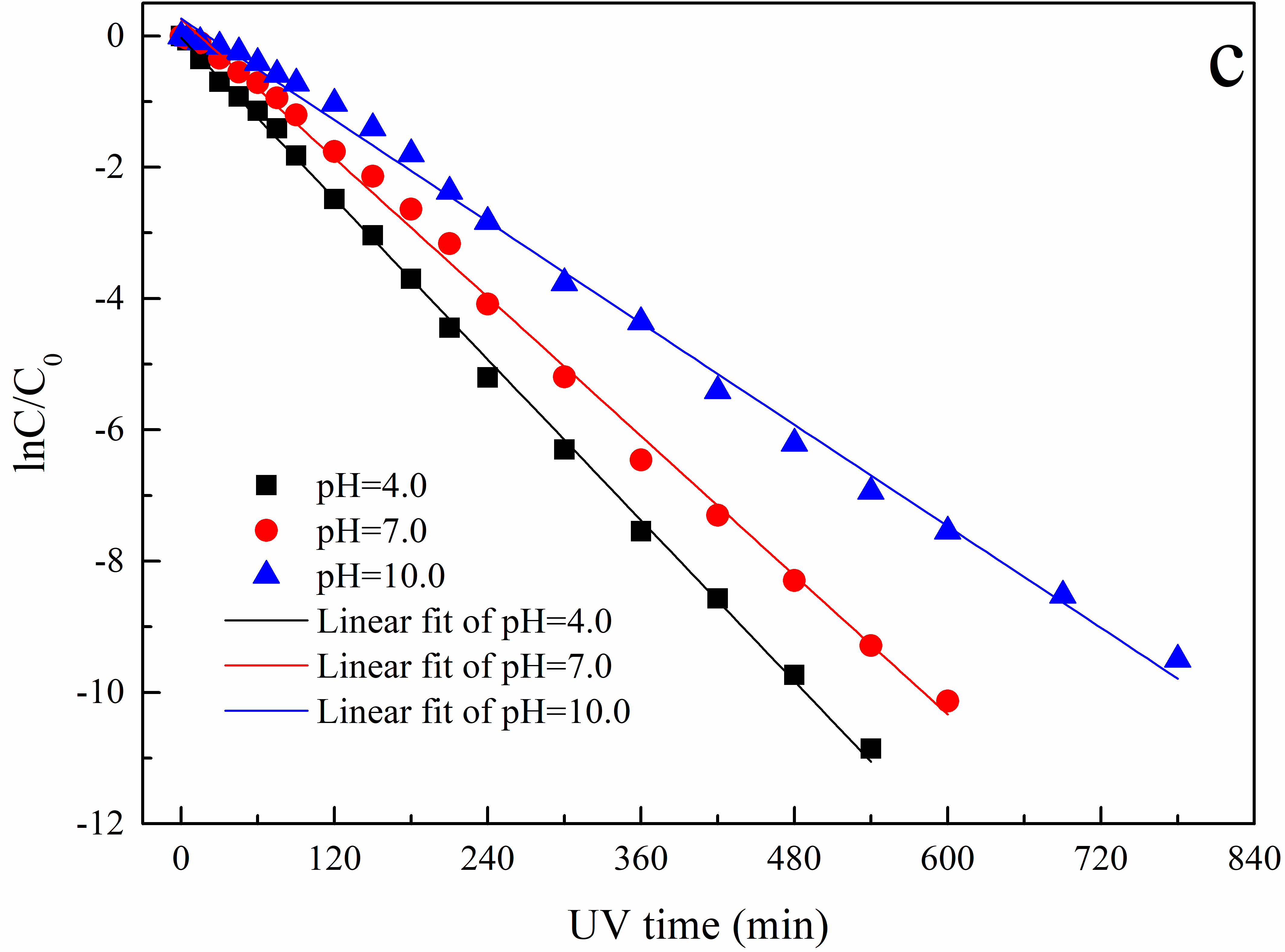


**Fig. S2** The fitting of different solution pH values for ATZ to a pseudo-first order kinetic model: (a) UV process; (b) UV/H_2_O_2_ process; (c) UV/TiO_2_ process.

**P1** (4-Isopropylamino-6-amino-*s*-triazine)

ESI^+^ mode

**Fig. S3** Molecular structure and MS/MS spectrum of P1 (ESI^+^, CE=22 eV).

**P2** (2-Methoxy-4-methylamino-6-isopropylamino-*s*-triazine)

ESI^+^ mode

**Fig. S4** Molecular structure and MS/MS spectrum of P2 (ESI^+^, CE=22 eV).

**P3** (2-Hydroxy-4-isopropylamino-6-vinylamino-*s*-triazine)

ESI^+^ mode

**Fig. S5** Molecular structure and MS/MS spectrum of P3 (ESI^+^, CE=0 eV).

**P4** (2-Hydroxy-4-ethylamino-6-isopropylamines-*s*-triazine)

ESI^+^ mode

ESI^-^ mode

**Fig. S6** Molecular structure and MS/MS spectrum of P4 (ESI^+^ and ESI^-^, CE=23 eV).

**P5** (2-Hydroxy-4-acetamido-6-ethylamino-*s*-triazine)

ESI^+^ mode

ESI^-^ mode

**Fig. S7** Molecular structure and MS/MS spectrum of P5 (ESI^+^ and ESI^-^, CE=16 eV).

**P6** (2-Hydroxy-4-(2-hydroxy-ethylamino)-6-vinylamino-*s*-triazine)

ESI^+^ mode

ESI^-^ mode

**Fig. S8** Molecular structure and MS/MS spectrum of P6 (ESI^+^ and ESI^-^, CE=24 eV).

**P7** (2-Hydroxy-4-acetamido-6-isopropylamino-*s*-triazine)

ESI^+^ mode

**Fig. S9** Molecular structure and MS/MS spectrum of P7 (ESI^+^, CE=26 eV).

**P8** (2-Methoxy-4-isopropylamino-6-ethylamino-*s*-triazine)

ESI^+^ mode

**Fig. S10** Molecular structure and MS/MS spectrum of P8 (ESI^+^, CE=22 eV).

**P9** (2-Chloro-4-ethylamino-6-isopropylamino-*s*-triazine)

ESI^+^ mode

**Fig. S11** Molecular structure and MS/MS spectrum of P9 (ESI^+^, CE=22 eV).

**P10** (2-Hydroxy-4-vinylamino-*s*-triazine)

ESI^+^ mode

**Fig. S12** Molecular structure and MS/MS spectrum of P10 (ESI^+^, CE=12 eV).

**P12** (2-Hydroxy-4-acetamido-6-isopropenylenylamino-*s*-triazine)

ESI^+^ mode

**Fig. S13** Molecular structure and MS/MS spectrum of P12 (ESI^+^, CE=20 eV).

**P13** (4-acetamido-*s*-triazine)

ESI^+^ mode

**Fig. S14** Molecular structure and MS/MS spectrum of P13 (ESI^+^, CE=15 eV).

**P14** (2-Hydroxy-4-acetamido-6-(2-hydroxyisopropylamino)-*s*-triazine)

ESI^+^ mode

ESI^-^ mode

**Fig. S15** Molecular structure and MS/MS spectrum of P14 (ESI^+^ and ESI^-^, CE=20 eV).

**P15** (2-Hydroxy-4-ethylimine-6-(2-hydroxyisopropylamino)-*s*-triazine)

ESI^+^ mode

ESI^-^ mode

**Fig. S16** Molecular structure and MS/MS spectrum of P15 (ESI^+^ and ESI^-^, CE=20 eV).

**P16** (2-Chloro-4-vinylamino-6-acetamido-*s*-triazine)

ESI^+^ mode

**Fig. S17** Molecular structure and MS/MS spectrum of P16 (ESI^+^, CE=15 eV).

**P17** (2-Hydroxy-4-(2-hydroxy-ethylamino)-6-isopropylamino-*s*-striazine)

ESI^+^ mode

**Fig. S18** Molecular structure and MS/MS spectrum of P17 (ESI^+^, CE=18 eV).

**P18** (2-Hydroxy-4-ethylamino-6-(2-hydroxyisopropylamino)-*s*-triazine)

ESI^+^ mode

**Fig. S19** Molecular structure and MS/MS spectrum of P18 (ESI^+^, CE=17 eV).

**P19** (2-Hydroxy-4-ethylamino-6-isopropenylenylamino-*s*-triazine)

ESI^+^ mode

**Fig. S20** Molecular structure and MS/MS spectrum of P19 (ESI^+^, CE=15 eV).

**P20** (2-Hydroxy-4-(2-hydroxy-ethylamino)-6-amino-*s*-triazine)

ESI^+^ mode

ESI^-^ mode

**Fig. S21** Molecular structure and MS/MS spectrum of P20 (ESI^+^ and ESI^-^, CE=20 eV).

**P21** (2-Chloro-4-vinylamino-6-amino-*s*-triazine)

ESI^+^ mode

ESI^-^ mode

**Fig. S22** Molecular structure and MS/MS spectrum of P21 (ESI^+^ and ESI^-^, CE=20 eV).
